# Exploring the chemical space of the lysine-binding pocket of the first kringle domain of hepatocyte growth factor/scatter factor (HGF/SF) yields a new class of inhibitors of HGF/SF-MET binding†
†Electronic supplementary information (ESI) available: Description of fragment library screening and data analysis, compound soaking, X-ray data collection and processing, molecular docking and biological assays. Crystallographic and refinement statistics. See DOI: 10.1039/c5sc02155c


**DOI:** 10.1039/c5sc02155c

**Published:** 2015-07-31

**Authors:** A. G. Sigurdardottir, A. Winter, A. Sobkowicz, M. Fragai, D. Chirgadze, D. B. Ascher, T. L. Blundell, E. Gherardi

**Affiliations:** a Department of Biochemistry , University of Cambridge , 80 Tennis Court Road , Cambridge , CB2 1GA , UK . Email: tlb20@cam.ac.uk ; Email: sigurdar@mrc-lmb.cam.ac.uk; b Medical Research Council (MRC) Center , Hills Road , Cambridge , CB2 0QH , UK; c Magnetic Resonance Center (CERM) and Department of Chemistry , University of Florence , Via L. Sacconi 6, 50019 Sesto Fiorentino , Florence , Italy; d Unit of Immunology and General Pathology , Department of Molecular Medicine , University of Pavia , 9 via A Ferrata , 27100 Pavia , Italy

## Abstract

The growth/motility factor hepatocyte growth factor/scatter factor (HGF/SF) and its receptor, the tyrosine kinase MET, constitute a signalling system essential for embryogenesis and for tissue/organ regeneration in post-natal life. HGF/SF-MET signalling, however, also plays a key role in the onset of metastasis of a large number of human tumours. Both HGF/SF and MET are high molecular weight proteins that bury an extensive interface upon complex formation and thus constitute a challenging target for the development of low molecular weight inhibitors. Here we have used surface plasmon resonance (SPR), nuclear magnetic resonance (NMR) and X-ray crystallography to screen a diverse fragment library of 1338 members as well as a range of piperazine-like compounds. Several small molecules were found to bind in the *lysine-binding pocket* of the kringle 1 domain of HGF/SF and its truncated splice variant NK1. We have defined the binding mode of these compounds, explored their biological activity and we show that selected fragments inhibit MET downstream signalling. Thus we demonstrate that targeting the *lysine-binding pocket* of NK1 is an effective strategy to generate MET receptor antagonists and we offer proof of concept that the HGF/SF-MET interface may be successfully targeted with small molecules. These studies have broad implications for the development of HGF/SF-MET therapeutics and cancer treatment.

## Abstract 2

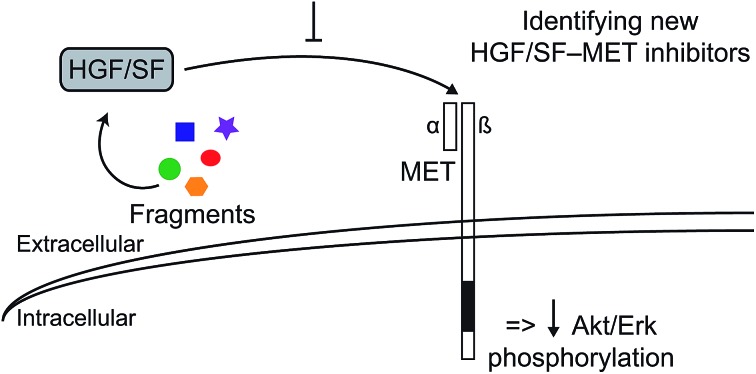
Targeting the *lysine-binding pocket* of the first kringle domain of HGF/SF using a fragment-based approach identified new chemical entities that can inhibit MET signalling.

## Introduction

Hepatocyte growth factor/scatter factor (HGF/SF)^1^,^2^ is a growth and motility factor essential for embryogenesis,^3^,^4^ liver regeneration^5^,^6^ and the repair of skin wounds.^7^ Binding of HGF/SF to its receptor, the MET tyrosine kinase,^8^ activates several signalling pathways including Ras/MAPK, PI3K/Akt and JAK/STAT.^9^,^10^ HGF/SF and MET also play crucial roles in human cancer where they control survival, growth and migration of tumour cells leading to metastasis.^9^,^10^ Abnormal MET signalling in human cancer is due to activating mutations^11^ or, more frequently, to over-expression of either ligand or receptor.^12^,^13^

**Fig. 1:**
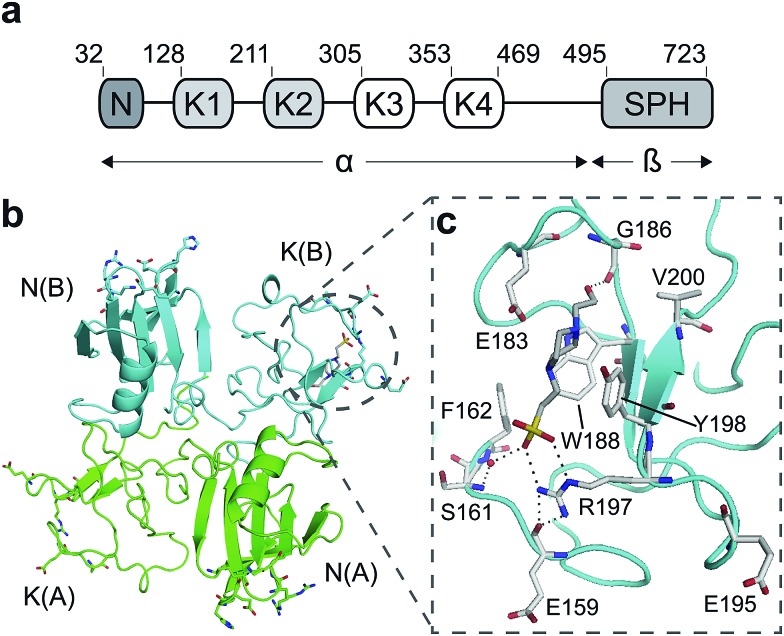
HGF/SF and its NK1 splice variant. (a) Domain structure of HGF/SF. The N-domain and the first kringle domain form NK1, a receptor agonist. Domains N with K1 to K4 form the α-chain of mature, activated HGF/SF. The α-chain is covalently linked to the β-chain, which consists of a serine-proteinase homology domain (SPH). (b) Crystal structure of the NK1 head-to-tail dimer (PDB accession: 1BHT 21). Protomers are labelled A (green) and B (cyan), the N- and K-domains are indicated and the *lysine-binding pocket* is encircled. (c) The *lysine-binding pocket* of the kringle domain of NK1 is formed by a glycine loop at the top of pocket (E183 and G186), an aromatic “base” (W188) and sides (F162 and Y198), as well as a positively charged anchor at the bottom of the pocket (R197). Main-chain segments are shown in cyan, side chain carbon atoms are in light gray, oxygen atoms are in red and nitrogen atoms in blue. A HEPES molecule is bound in the pocket and shown with its carbon atoms in dark gray and its sulphur atom in yellow. Hydrogen bonds between the HEPES molecule and NK1 are represented by dashed lines. (In relationship to (b), (c) is rotated 180° around the *x*-axis.)

HGF/SF has a domain structure and proteolytic mechanism of activation similar to those of the blood proteinase precursor plasminogen.^14^ The inactive precursor form of HGF/SF is cleaved at a trypsin-like site located between the fourth kringle (K) and the C-terminal domain. Cleavage produces an active, disulphide-linked, two-chain protein^15^ consisting of a 69 kDa α-chain and a 34 kDa β-chain which is homologous to the catalytic domains of serine proteinases (SPH domain) (Fig. 1a).^14^ Two truncated forms of HGF/SF are produced by alternative splicing of the primary transcript: NK1 encodes the N domain and the first K domain (K1),^16^ while NK2 encodes the N, K1 and K2 domains.^17^ Both NK1 and NK2 were initially described as receptor antagonists, but experiments in transgenic mice have demonstrated unequivocally that NK1 behaves *in vivo* as a partial receptor agonist.^18^ NK1 is a monomer in solution, but crystallises as a head-to-tail dimer (Fig. 1b) and all available crystal structures of human and mouse NK1 yielded the same dimer.^19^–^22^

The key role of HGF/SF-MET signalling in metastasis has propelled MET among the top targets for human cancer therapy^23^ and substantial progress has been made in recent years in developing inhibitors of the MET kinase and antibodies directed to HGF/SF or the MET ectodomain. However, there are potential limitations to both approaches. Resistance to kinase inhibitors develops rapidly through mutations^24^ and antibodies – which may not be hampered by the development of resistance – may prove prohibitively expensive for the large cancer patient groups in which MET is involved.

Here we propose and explore a novel strategy toward MET therapeutics, namely small molecule receptor antagonists. These molecules, may potentially overcome the limitations of the kinase inhibitors and antibodies, and may have a further advantage over kinase inhibitors, namely the fact that their site of action is extracellular.

HGF/SF binds the N-terminal β-propeller domain of MET, utilising two binding sites: a high-affinity site located within the NK1 fragment and a low-affinity one contained within the SPH domain.^25^–^28^ Mutagenesis studies identified several residues clustered in solvent exposed areas of the K domain of NK1 (E159, S161, E195, R197 and Y198) which are involved in receptor binding or activation^28^ (Fig. 1b). This patch of residues is surrounded by a pocket,^19^,^21^ similar to the one shown to bind lysine in the K domains of other proteins.^29^,^30^ Three of seven residues that contribute to the binding pocket in the K4 domain of plasminogen are not conserved in the one present in the K1 domain of HGF/SF, and a glycine residue (G185) is inserted in the loop that borders one side of pocket, resulting in a substantial change in conformation. These differences may not enable binding of lysine side chains or lysine analogues but we retain the name ‘*lysine-binding pocket*’ throughout the present work in order to acknowledge the structural homology.

A HEPES (4-(2-hydroxyethyl)-1-piperazineethanesulfonic acid) molecule was found bound into this pocket in several crystal structures of NK1 (ref. 20–22) suggesting affinity for sulphated molecules *in vivo*. We hypothesised that the *lysine-binding pocket* of K1 of HGF/SF could offer a way to target the HGF/SF-MET interface and undertook a fragment screen in order to test this hypothesis. Here we report that several small molecules bind into this pocket and can inhibit MET signalling. Thus we provide proof of concept for a new class of MET inhibitors for cancer therapy, namely small-molecule receptor antagonists.

## Results

### Piperazine-like compounds bind into the *lysine-binding pocket* of HGF/SF

Several crystal structures of NK1, for example 1BHT,^21^ 1GMN^20^ and 1GP9,^22^ have shown a HEPES molecule bound in the *lysine-binding pocket* of the K1 domain. It has also been observed that the formation of NK1-MET complexes was inhibited in buffers containing 50 mM HEPES (Lauris Kemp, personal communication). The pocket is elliptical in shape with a total volume of 211 Å^3^. It is made up of a largely aromatic “base” (W188), aromatic pocket sides (F162 and Y198), an entrance bracketed by a positively charged surface at one end (R181 and R197) and a neutral glycine loop involving G185 and G186 at the opposite end (Fig. 1b and c). These features make the pocket quite complex in nature and attractive from the point of view of drug development.

In initial experiments HEPES was successfully incorporated into apo NK1 crystals by soaking and bound into the *lysine-binding pocket* in a mode consistent with previous results. Next, three classes of piperazine-like sulphonated compounds containing piperazine ((H)EPPS and PIPES), morpholine (MES and MOPS), or cyclohexane (CHES and CAPS) rings were studied for their binding to NK1 (Table 1), alongside another five sulphonated compounds selected from a chemical fragment library (Table 2). All compounds were soaked into NK1 apo crystals that diffracted to maximum resolutions ranging from 1.9 to 2.5 Å; the space group was determined to be *P*2_1_ in all cases, with unit cell parameters comparable to those of the apo crystals. For crystallographic statistics, see ESI Tables S1 and S2.† Fig. S1† shows representative examples of the electron density of the *lysine-binding pocket* for NK1 in complex with the piperazine-like compounds.

**Table 1:** Compounds selected for soaking into NK1 apo crystals. Piperazine-like compounds with a sulphonic acid group and their binding constants obtained using SPR and NMR.

Piperazine-like compounds	Structure	*K*_D_ in SPR^*a*^ [mM]	*K*_D_ in NMR^*a*^ [mM]
HEPES	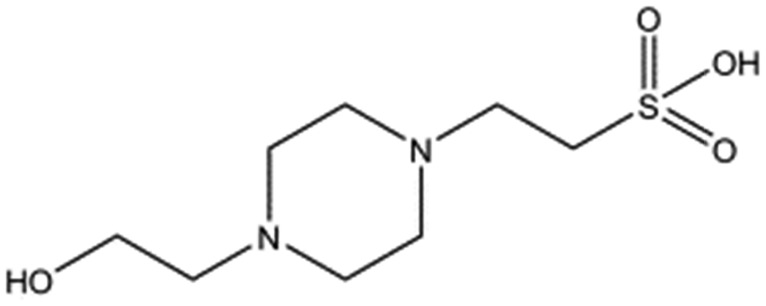	2.4 ± 0.4	3.3 ± 0.4
(H)EPPS	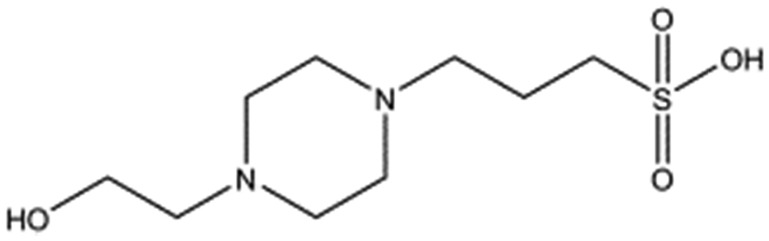	7.0 ± 1.0	4.4 ± 1.8
PIPES	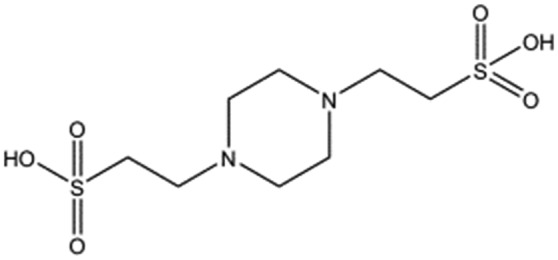	26.6 ± 0.6	45.9 ± 4.7
MES	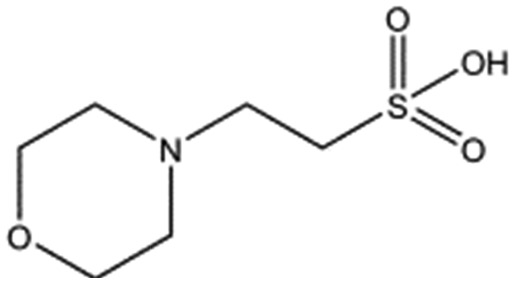	24.3 ± 3.1	13.4 ± 5.7
MOPS	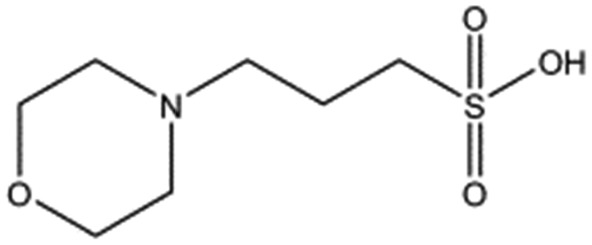	7.0 ± 1.0	4.7 ± 1.7
CHES	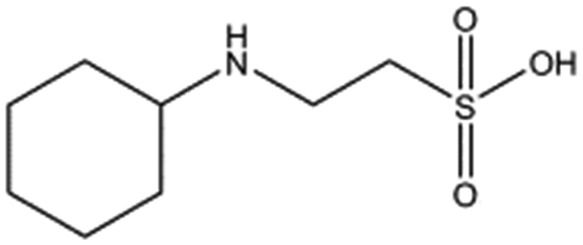	30.2 ± 3.1	15.7 ± 4.5
CAPS	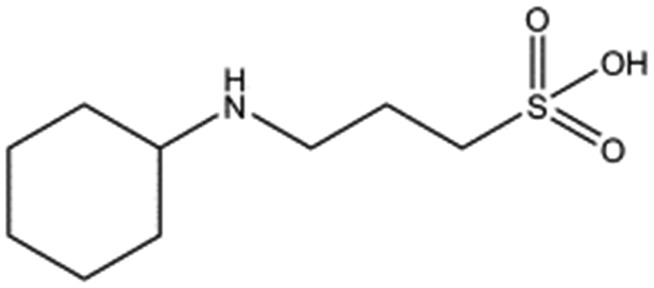	1.2 ± 0.2	1.4 ± 0.5

^*a*^Steady-state binding constants.

**Table 2:** Compounds selected for soaking into NK1 apo crystals. Small compounds that have a sulphonic acid group.

Compound name		Structure	
Taurine		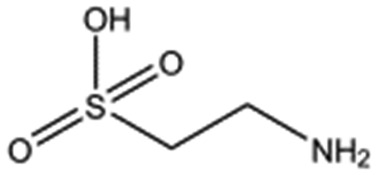	
3-Hydroxypropane-1-sulphonic acid (2FA)		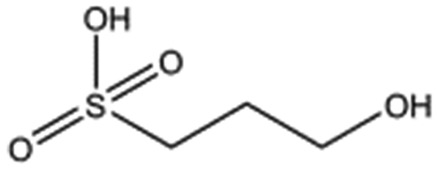	
*p*-Toluene sulphonic acid		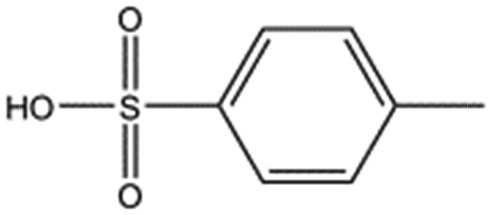	
Propargyl benzene sulphonate		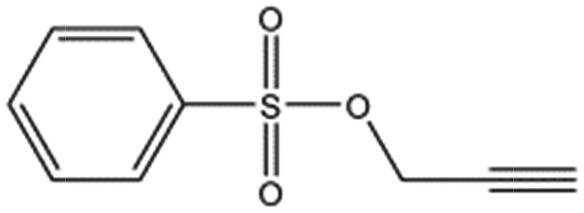	
3-Amino-1-propane sulphonic acid		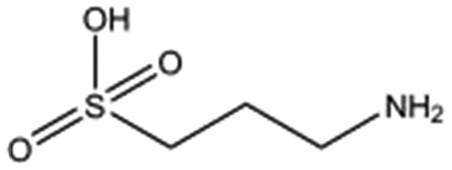	

**Fig. 2:**
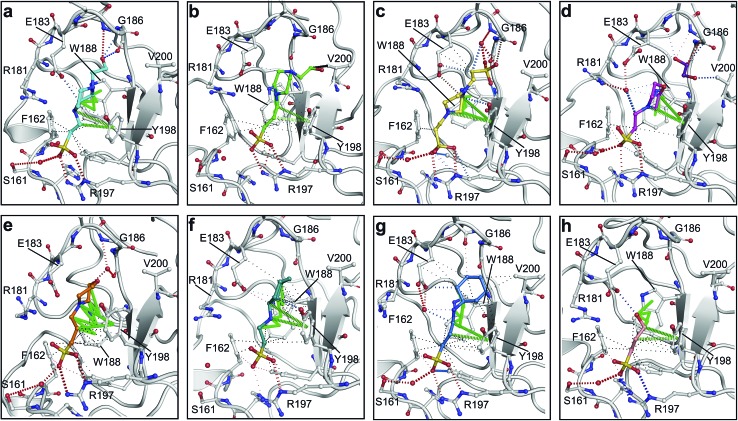
The piperazine-like compounds bind in the *lysine-binding pocket* of the kringle domain of NK1. (a) HEPES (cyan, PDB ID: ; 5COE), (b) (H)EPPS (green, PDB ID: ; 5CS3), (c) PIPES (yellow, PDB ID: ; 5CS5), (d) MES (magenta, PDB ID: ; 5CS9) (and an ethylene glycol molecule (purple)), (e) MOPS (orange, PDB ID: ; 5CSQ), (f) CHES (blue green, PDB ID: ; 5CT1), (g) CAPS (light blue, PDB ID: ; 5CT2) and (h) 2FA (cyan, PDB ID: ; 5CT3), bound in the *lysine-binding pocket* of NK1 (light gray). Side chain atoms of residues and water molecules (dark red) involved in binding the compounds are shown. Hydrogen bonds are represented by red dashed lines, polar interactions by blue dashed lines, hydrophobic interactions by black dashed lines and π-interactions by green dashed lines. Weak interactions are shown in a lighter colour and stronger interactions by a darker colour. Protein carbon atoms are gray, oxygen atoms are in red and nitrogen atoms are in blue.

All piperazine-like molecules bound into the *lysine-binding pocket* of NK1 (Fig. 2) and an overlay of the three classes of piperazine-like sulphonated compounds bound to NK1 is shown in ESI Fig. S2.† HEPES forms two crucial hydrogen bonds to R197 through the sulphonic acid group (distance 2.7–2.8 Å) and one main chain hydrogen bond to S161 through a water molecule. The piperazine moiety of HEPES makes strong carbon and donor π-interactions with W188 and Y198, and weak polar interactions with E183, whereas the hydroxyl group engages the glycine loop in weak polar interactions (Fig. 2a). While the binding mode of (H)EPPS is very similar to that of HEPES, the propane sulphonic acid group rotates and positions the piperazine ring closer to the entrance of the pocket, imperfectly stacked against Y198. The compound is unable to make a hydrogen bond to G186 *via* its hydroxyl group, but instead forms a hydrophobic interaction with V200 (Fig. 2b). PIPES contains ethane sulphonic acid groups on either side of the piperazine moiety, one of which makes the conserved hydrogen bonds to R197 while the other forms a hydrogen bond to G186 and interacts further with the glycine loop through a water molecule. The length of the compound causes an orientation of the sulphonic acid group towards R197 which is slightly different from the ones observed with HEPES and (H)EPPS: the piperazine moiety is positioned deeper into the cavity and the weaker electron density of the compound around G186 suggests a degree of flexibility.

MES, MOPS, CHES and CAPS bind the *lysine-binding pocket* of NK1 in the same way as HEPES, with their sulphonic groups forming two hydrogen bonds to R197 and one to S161 mediated by a water molecule (except CHES) (Fig. 2d–g). However, unlike HEPES, MES does not occupy the pocket around the glycine loop, allowing an ethylene glycol molecule to occupy the space and form a hydrogen bond to G186 (Fig. 2d). CAPS and CHES contain an additional nitrogen atom in the sulphonic acid chain, extending this part of the molecule. CAPS has the longest chain of all the piperazine-like compounds, which positions the cyclohexane ring further away from R197 (Fig. 2g). This longer chain enables CAPS to interact with a larger surface area of the pocket, including E183 and V200 through hydrophobic interactions. MOPS is a propane sulphonic acid compound like (H)EPPS and its morpholine group is positioned in a similar position to the piperazine group of (H)EPPS (Fig. 2e). The overlaid structures of MOPS and (H)EPPS align well. The extra carbon in the chain of MOPS allows more π-interactions to W188 compared to MES and in addition the morpholine ring is able to interact with the carbonyl oxygen of G186 through a water molecule.

NK1 crystals soaked with the sulphonated compounds (Table 2) diffracted to a maximum resolution ranging from 1.9 to 2.4 Å and the space group was determined to be *P*2_1_ in all cases, with unit cell parameters comparable to those previously collected for apo NK1 crystals. Only one compound however, 3-hydroxypropane-1-sulphonic acid (2FA, Fig. 2h), was bound in the *lysine-binding pocket* (crystallographic statistics are listed in ESI Tables S1 and S2†). The binding mode for 2FA is similar to HEPES: the sulphonic acid group forms two hydrogen bonds to R197 and a hydrogen bond to S161 through a water molecule. In addition, 2FA makes polar interactions with E183 *via* its hydroxyl group and π-interactions with W188 and Y198.

**Fig. 3:**
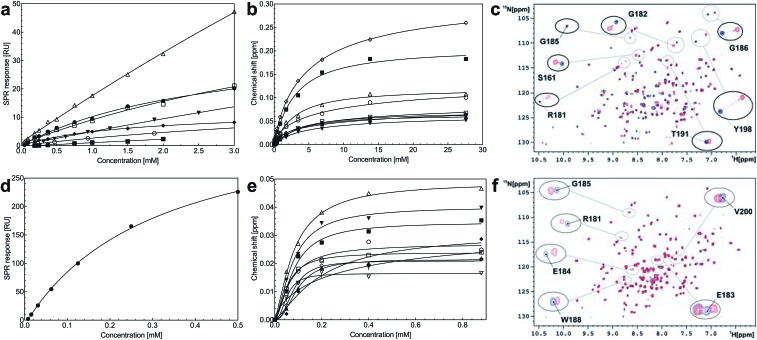
Binding affinities of the piperazine-like compounds and MB605 for the *lysine-binding pocket* of the kringle domain of NK1. (a) Steady-state binding curves for the piperazine-like compounds from SPR. HEPES ([black circle]), (H)EPPS (○), PIPES ([blacktriangledown]), MES (△), MOPS (□), CHES (■) and CAPS (♦). (b) Binding curves of the residues that experience the largest chemical shift in 2D ^1^H-^15^N HSQC NMR spectroscopy upon binding of HEPES to NK1. S161 ([black circle]), R181 (○), E183 ([blacktriangledown]), E184 (△), G185 (■), W188 (□), R197 (♦), Y198 ([square tilted open]) and V200 (▲). (c) Overlaid 2D ^1^H-^15^N HSQC-spectra of ^15^N-enriched NK1 in the absence (blue) and presence of 0.9 mM HEPES (red). Selected residues of the *lysine-binding binding* pocket are highlighted showing the chemical shift upon binding. (d) Steady-state binding curve of MB605 ([black circle]) to immobilized NK1 measured by SPR. (e) Binding curves of the residues that experience the largest chemical shift upon binding of MB605 to NK1. K144 (|), R181 (○), E183 ([blacktriangledown]), E184 (△), G185 (■), W188 (□), R197 (♦), E199 ([square tilted open]), V200 (▲) and C201 (▽). (f) Overlaid 2D ^1^H-^15^N HSQC spectra of ^15^N enriched NK1 in the absence (blue) and presence (red) of a ligand (MB605 440 μM). Selected residues of the *lysine-binding pocket* are highlighted showing the chemical shift upon binding.

Steady-state binding constants of the seven piperazine-like compounds were determined using SPR (Fig. 3a and Table 1) and NMR (Fig. 3b and c and Table 1). In NMR experiments, chemical shift perturbations of selected residues of the *lysine-binding pocket* could be readily observed upon binding of the compounds. Y198 and G185 showed the largest variations in chemical shift upon binding, and binding constants were obtained by titration of the compounds into the protein sample (representative data with HEPES are shown in Fig. 3b and c, for other piperazine-like compounds see ESI Fig. S3†). Binding constants obtained from SPR and NMR are comparable (Table 1) and the figures indicate that CAPS and HEPES display the highest binding affinities, whereas PIPES and CHES display the lowest. The affinity data are well rationalised by differences in the chain length of the compounds and by the different modes of binding revealed by X-ray crystallography (see Discussion).

### Screening of a fragment library identifies new chemical entities binding into the lysine-binding pocket of the kringle 1 domain of HGF/SF

A screen of a fragment library of 1338 fragments for NK1 binders was carried out by SPR and yielded 71 confirmed hits (see ESI Materials & Methods and ESI Fig. S4† for full details of the screen). The 71 compounds could be classified into five main scaffolds: benzene ring (23), 6-membered heterocyclic derivatives (12), 5-membered heterocyclic derivatives (22), bicyclic derivatives (7), and two connected rings (7) (see ESI Tables S3–S7† for a full list of compounds).

Steady-state binding studies by SPR showed good binding behaviour and *K*_D_ < 3 mM for 24 fragments which are deemed validated hits (five of which contain a carboxylic acid group). 28 fragments displayed linear, concentration-dependent binding indicative of stacking on the chip surface and were therefore discarded. 11 fragments failed to yield reproducible binding. Ten out of the 24 validated hits had *K*_D_ < 1 mM (Table 3) with MB605 being the best fragment hit with a *K*_D_ value of 310 ± 40 μM (Fig. 3d and Table 3). NMR measurements (^1^H-^15^N TROSY-HSQC) were carried out to assess binding of selected compounds out of the original 71 hits to NK1. These represent hits with different steady-state affinities: MB605, AT0381 and MB895 displaying good affinities at <1 mM, MB1299, MB389 and MB915 medium steady-state affinities (1–4.5 mM) and MB1283 the lowest affinity. Only MB605 showed variation in chemical shifts for several NK1 residues, mainly in and around the *lysine-binding pocket*, for example E183, R181 and E184, yielding a *K*_D_ of 110 μM ± 10 μM (Fig. 3e and f) that compares well with the value obtained by SPR (Fig. 3d and Table 3).

**Table 3:** The ten fragments obtained from fragment screening that showed affinities better than 1 mM: structures, molecular weights, response levels from the initial fragment screening and respective *K*_D_ values

Compound name	Structure	Molecular weight [Da]	SPR response^*a*^ [%]	*K*_D_ ^*b*^ [mM]
MB605	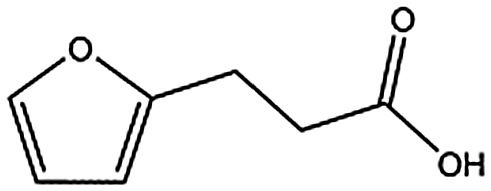	140.1	215	0.31 ± 0.04
MB1261	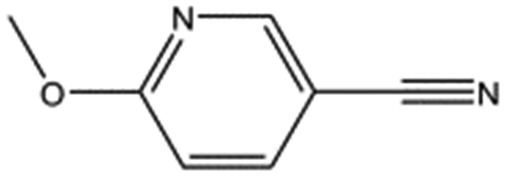	134.1	95	0.35 ± 0.04
MB1318	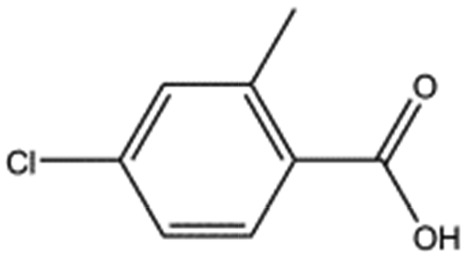	170.6	61	0.36 ± 0.04
MB1082	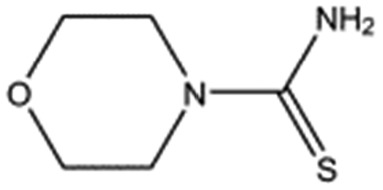	146.2	53	0.37 ± 0.04
MB417	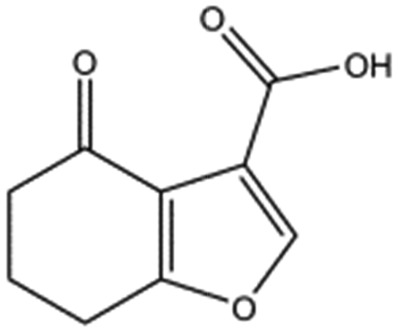	180.2	87	0.42 ± 0.04
MB895	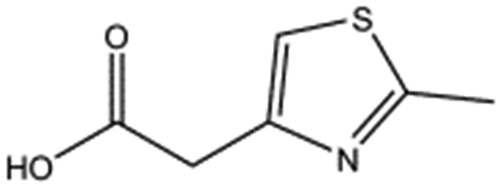	157.2	53	0.43 ± 0.05
AT0381	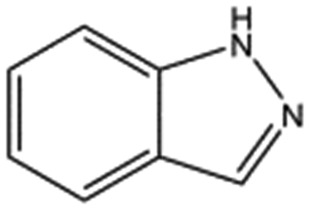	118.1	53	0.74 ± 0.06
CA023	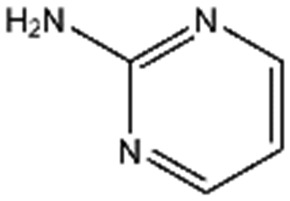	95.1	52	0.77 ± 0.06
MB1284	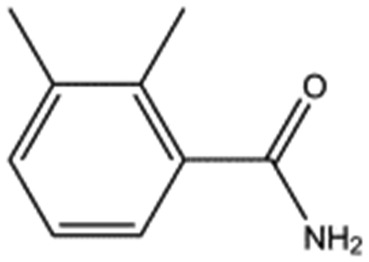	149.2	90	0.86 ± 0.07
MB1315	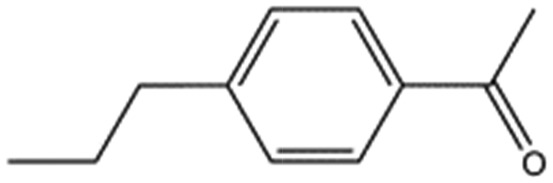	162.2	52	0.94 ± 0.09

^*a*^Normalised relative value against NK1.

^*b*^Steady-state binding constant from SPR.

**Fig. 4:**
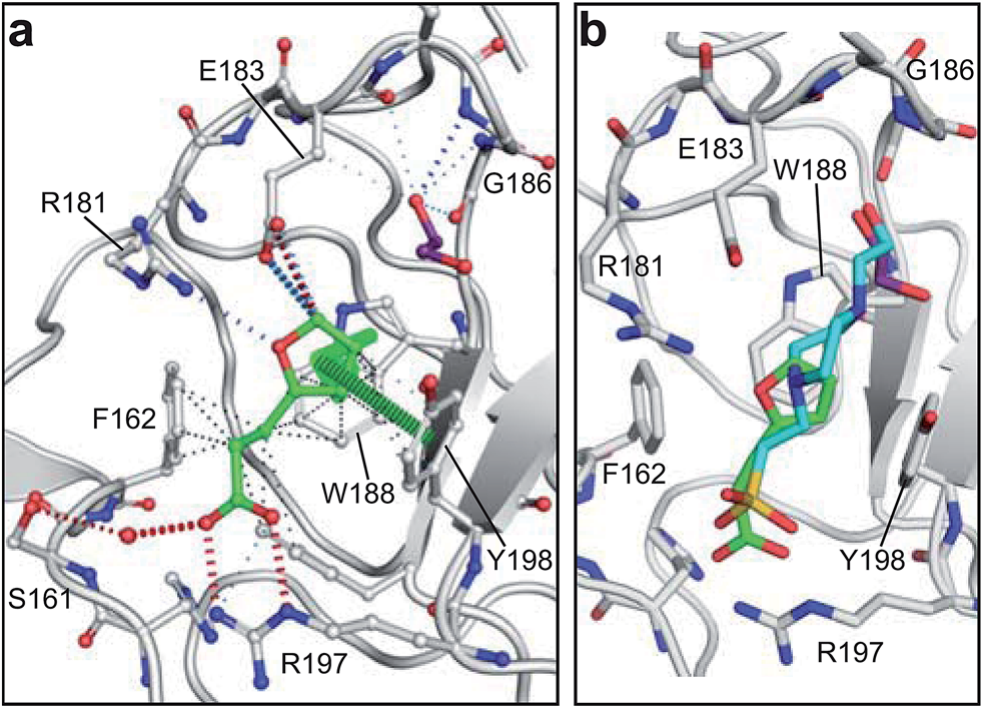
Binding of MB605 in the *lysine-binding pocket* of the kringle domain of NK1. (a) MB605 (green, PDB ID: ; 5CP9) binds in the *lysine-binding pocket* with a binding mode similar to HEPES but forms additional hydrogen bonds to R181 and E183 accounting for a higher affinity. An ethylene glycol molecule (purple) is bound in the NK1_MB605 structure filling the top of the pocket. Hydrogen bonds are represented by red dashed lines, polar interactions by blue dashed lines, hydrophobic interactions by black dashed lines and π-interactions by green dashed lines. Weak interactions are shown in a lighter colour and stronger interactions by a darker colour. (b) Comparison of the binding mode of MB605 (green) and HEPES (cyan). Side chain atoms of residues and a water molecule (dark red) involved in binding are shown. Protein carbon atoms are gray, oxygen atoms are in red and nitrogen atoms are in blue.

MB605 consists of two parts, a furan and a propanoic acid moiety, resembling HEPES. A crystal structure of MB605 soaked into NK1 apo crystals (see ESI Tables S1 and S2† for crystallographic statistics) demonstrated a binding mode similar to HEPES in which the carboxylic acid group of MB605 forms two hydrogen bonds to R197 and one main-chain hydrogen bond to S161 through a water molecule. In comparison to HEPES, MB605 forms stronger polar interactions and hydrogen bonds to E183 *via* the furan ring, as well as additional polar interactions to R181. The furan ring packs tightly in the aromatic part of the pocket, stacking against W188 and resting perpendicular to Y198. In this orientation, MB605 is able to make π–π interaction to W188 and Y198. Additionally, one molecule of the cryoprotectant ethylene glycol is bound in the *lysine-binding pocket*, 3.5 Å away from the furan ring, and makes hydrogen bonds to G185 and G186 (Fig. 4a), similar to the NK1_MES structure. An overlay of MB605 and HEPES is shown in Fig. 4b.

**Fig. 5:**
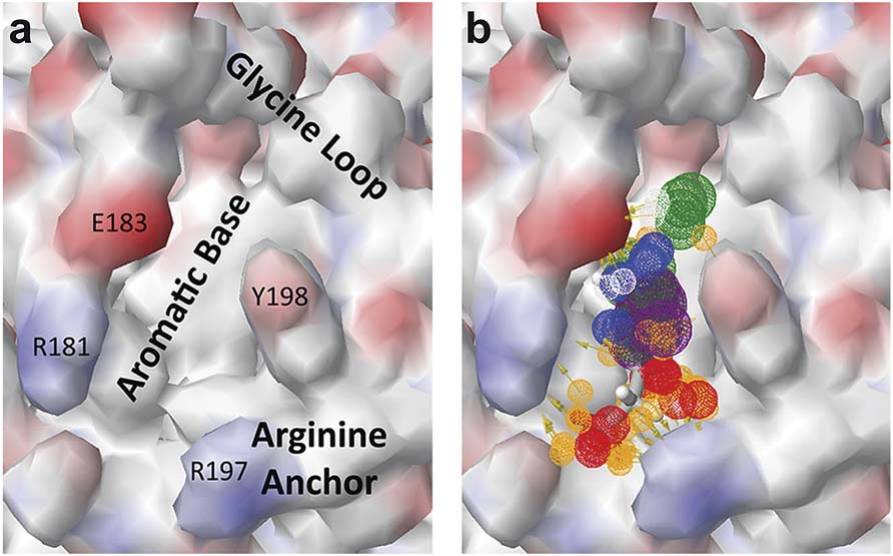
Pharmacophore model of the NK1 *lysine-binding pocket*, showing the important interactions made by the piperazine-like and fragment hits. (a) Key determinants of the shape and the chemical character of the *lysine-binding pocket* of the kringle domain of NK1. (b) The chemical characteristics of the small molecule binders are represented by mesh spheres, with green representing hydrogen donors and positive ions, purple aromatic groups, white and orange hydrogen donors and acceptors respectively, and blue and red positive and negative ions respectively.

The chemical structures of the nine other fragments with *K*_D_ values better than 1 mM (Table 3) resemble MB605 in that they all contain a ring and a functional group or atoms capable of forming two hydrogen bonds. While solubility issues prevented us from obtaining structures of these complexes, we explored possible binding modes in docking studies using GOLD.^31^,^32^ All compounds successfully docked into the *lysine-binding pocket* in a similar position to MB605 and docking scores and interactions were consistent with their experimental *K*_D_ values (data not shown). Pharmacophore analysis of the binding of the fragments and piperazine-like molecules within the *lysine-binding pocket* (Fig. 5) highlighted both the pivotal role of R197 in anchoring the small molecules within the pocket through hydrogen bonds and the importance of additional aromatic interactions made to Y198 and the aromatic base of the pocket.

We studied five additional, MB605-related compounds identified *via* a substructure search of the ZINC database of commercially available compounds, namely: (i) 2-thiophenepropanoic acid (CAS number 5928-51-8) differing from MB605 in having a sulphur atom in the 5-membered ring instead of oxygen, (ii) hydrocinnamic acid (501-520-0) harbouring a benzene ring instead of a furan, (iii) 2-phenylethyl phosphonic acid (4672-30-4), (iv) T5445870, structurally very similar to MB605 but containing an additional phenyl ring substituted at the C4 atom of the furan ring and (v) T5480685, a compound that could stack well against the indole group of W188 through its benzofuran core. Affinity values for these compounds were obtained by SPR and range from a *K*_D_ value of 0.7 to 2.2 mM (Table 4). Compound T5480685 had a *K*_D_ of 0.7 ± 0.2 mM and is the best obtained from this set of compounds, reflecting the possible favourable interaction of the benzofuran to W188. The decrease in affinity for compounds 501-52-0 and 4672-30-4 compared to MB605 (and 5928-51-8), demonstrates that a 5-membered heterocyclic ring is preferred over a benzene for binding in the aromatic part of the pocket. T5445870 is structurally very similar to MB605, but the additional phenyl ring is probably too large to occupy the space by the glycine loop, which results in a decreased the steady-state affinity of 2.1 ± 0.3 mM.

**Table 4:** Five compounds obtained based on MB605 substructure similarity: structures, molecular weights and respective *K*_D_ values

Compound name	Structure	Molecular weight [Da]	*K*_D_ ^*a*^ [mM]
5928-51-8	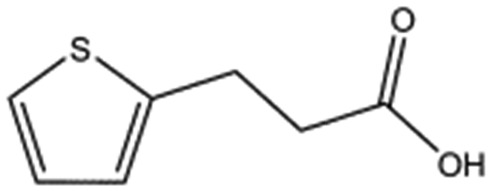	156.2	1.1 ± 0.3
501-520-0	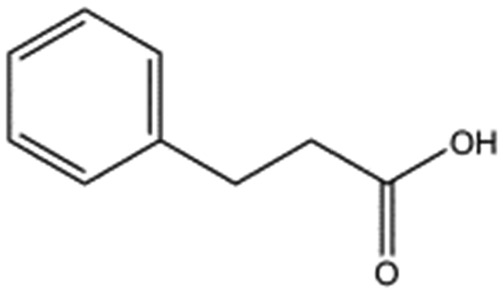	150.2	2.2 ± 0.9
4672-30-4	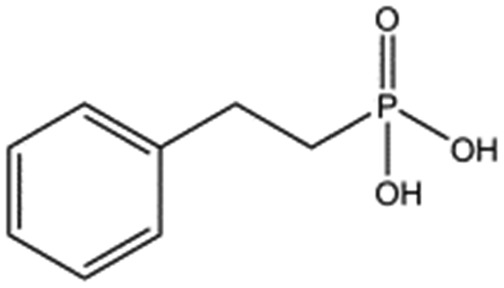	186.1	2.2 ± 0.4
T5445870	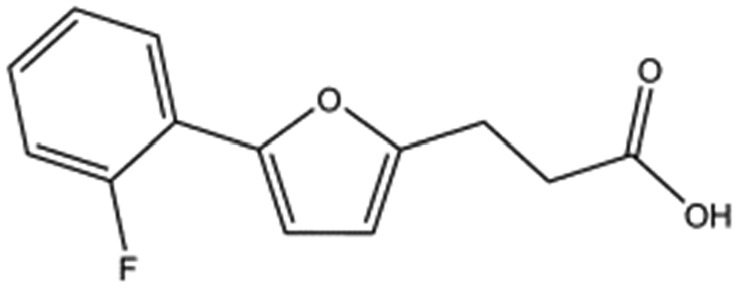	234.2	2.1 ± 0.3
T5480685	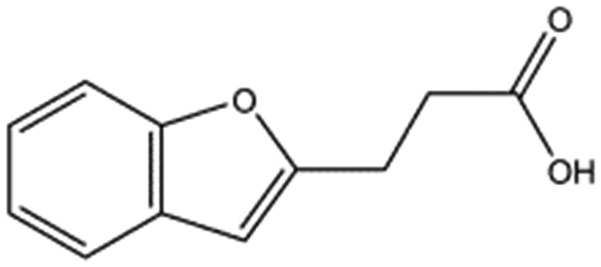	190.2	0.7 ± 0.2

^*a*^Steady-state binding constant from SPR.

### Compounds binding into the lysine-binding pocket of the K1 domain can inhibit NK1-induced MET activation

**Fig. 6:**
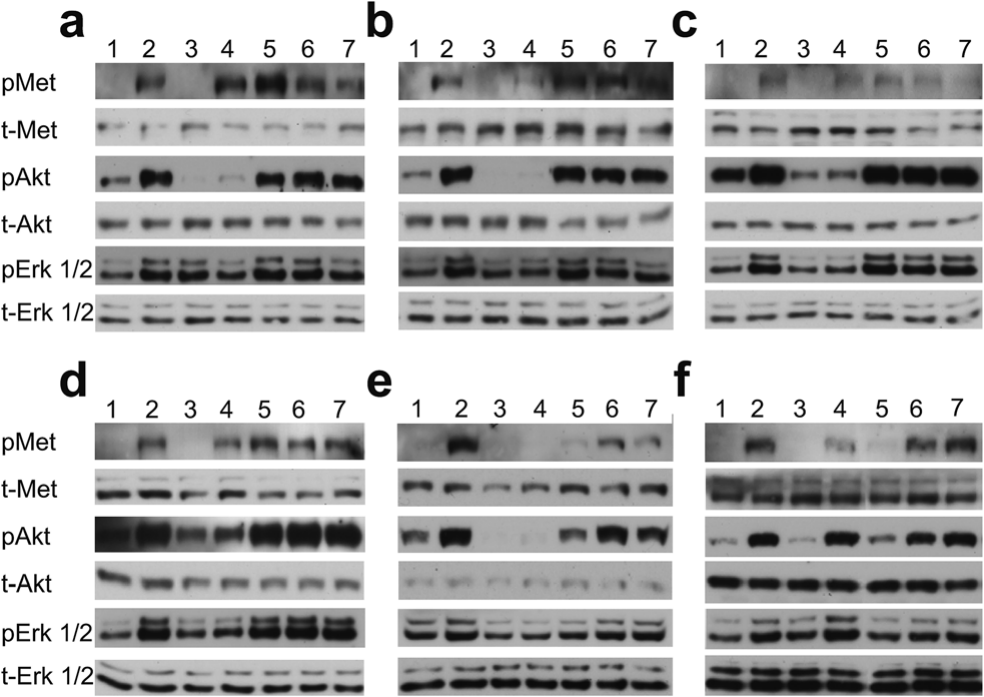
Biological activity of the piperazine-like compounds and MB605. (a–e) Vero cells expressing the receptor endogenously on their cell surface were stimulated with HGF/SF or NK1 and phosphorylation of MET and downstream targets Akt and Erk1/2 was analysed. Piperazine-like compounds HEPES (a), (H)EPPS (b), MES (c), MOPS (d) and CAPS (e) were tested at 100 mM (lanes 4), 10 mM (lanes 5), 1 mM (lanes 6) and 100 μM (lanes 7) concentration in the presence of 1 nM NK1. To control for activating or inhibiting effects of the compounds independently of MET, piperazine-like compounds were tested on cells at 100 mM without stimulation by NK1 (lanes 3). Biological activity of MB605 (f) was investigated at 1 mM (lane 5), 100 μM (lane 6) and 10 μM (lane 7) in the presence of a constant concentration of DMSO (0.1%). A solvent control (0.1% DMSO, lane 3) was included in order to account for the effect of DMSO on the cells. A positive control (1 nM NK1 in the presence of 0.1% DMSO) is shown in lane 4. In all experiments, un-stimulated cells served as negative control (lanes 1) while cells stimulated with 1 nM NK1 served as positive control (lanes 2).

We evaluated the effect of piperazine-like compounds and MB605 on Vero cells by assessing MET, Erk 1/2 and Akt phosphorylation in response to stimulation using 10 nM NK1. MB605 was dissolved in DMSO, which limited the range of concentrations tested to ≤1 mM in order to avoid solvent effects on cell function/viability. The water-soluble piperazine-like compounds, in contrast, could be tested at concentrations up to 100 mM. Inhibition of phosphorylation of Erk 1/2 and Akt (S473) was seen for HEPES, (H)EPPS, MES, MOPS and CAPS (Fig. 6a–e) at the highest concentration (100 mM, lanes 4). PIPES and CHES showed similar results (data not shown). CAPS was also active at a concentration of 10 mM (Fig. 6e, lane 5).

MB605 was tested at three concentrations, ranging from 1 mM to 10 μM (Fig. 6f). The compound was active at a concentration of 1 mM (lane 5), in line with the *K*_D_ values obtained by SPR and NMR (Table 3 and Fig. 3).

## Discussion

The *lysine-binding pocket* of NK1 is important for binding to and activation of MET, and our results show that compounds binding into this pocket offer a new way to target the HGF/SF-MET interface with small molecules. We used three different ways to target this interface: – a fragment screen, which identified 24 validated hits, – a similarity search based on the best fragment hit and, – the analysis of a series of molecules related to a known binder. Binding affinities of the compounds examined in this study ranged from 0.3 to 30 mM reflecting differences in their structure and their resulting binding. Specifically, we found that affinities increased when:

(I) a hydrogen bond acceptor (carboxylic or sulphonic acid) is able to anchor the molecule to R197,

(II) a hydroxyl group is able to form a hydrogen bond to G186,

(III) an alkyl chain or ring structure is present to connect the acid and hydroxyl group, which is able to fit into the aromatic pocket formed by W188, F162 and Y198 (distance from acid group to ring is three rather than two carbon atoms) and form π-interactions (π–π interactions increase the affinity even further).

All of the compounds discussed here, as confirmed by crystallographic analysis, show the conserved and crucial hydrogen bonds to R197 and the differences in binding affinity reflect additional interactions. This is demonstrated, for example, by HEPES (*K*_D_ = 2.4 mM) which, compared to (H)EPPS (*K*_D_ = 7.0 mM) and PIPES (*K*_D_ = 26.6 mM), forms stronger π-interactions to W188 an Y198, weak polar interactions to E183 and further contacts with the glycine loop. Similar arguments explain the difference in binding affinity between MOPS (*K*_D_ = 7.0 mM) and MES (*K*_D_ = 24.3 mM). CAPS (*K*_D_ = 1.2 mM), the longest of all the piperazine-like compounds, interacts *via* its cyclohexane ring with a larger surface of the pocket, including E183 and V200, making it the highest affinity piperazine-like compound. MB605 on the other hand has its furan ring in an ideal orientation to form strong π–π interactions with both W188 and Y198, in addition to making stronger polar interactions and hydrogen bonds to E183 than any of the piperazine-like compounds and an additional polar interaction to R181; yielding the highest affinity reported here (*K*_D_ = 310 μM).

These promising results clearly set the prospect of a new class of MET inhibitors targeting the *lysine-binding pocket* of the first kringle domain of HGF/SF, but further work is needed in order to increase the affinity and hence the potency of the compounds as well as to ensure target specificity. Kringle domains are present in a variety of proteins of the fibrinolytic and blood coagulation system including plasminogen,^33^ tissue plasminogen activator,^34^ urokinase plasminogen activator,^35^ and thrombin, as well as in several other proteins such as apo(a) and the ROR1/2 receptors. Despite the apparently uniform structural characteristics, the lysine-binding pocket of different kringle domains, are responsible for the binding specificity of the cognate proteins. For example, the affinity of human plasminogen and tissue plasminogen activator for fibrin appears to arise from interactions of selected kringle domains with lysine side chains exposed by the fibrin matrix.^36^ The *lysine-binding pocket* of the first kringle domain of HGF/SF has a distinct shape and a strong aromatic character, which sets it apart from the corresponding pockets of the kringle domains of other proteins.^21^ Indeed, in spite of clear similarities between the binding of HEPES to the *lysine-binding pocket* of the first kringle of HGF/SF (Fig. 2a), and the binding of lysine analogues, *i.e.* the anti-fibrinolytic agents ε-aminocaproic acid (EACA) and *trans*-4-(aminomethyl)cyclohexane-1-carboxylic acid (AMCHA) to plasminogen kringles,^37^ we have found that neither EACA or AMCHA binds NK1 (A. G. S., unpublished data). These results clearly demonstrate that the lysine-binding pockets of the kringle domains of different proteins offer a way to develop protein-specific inhibitors, a concept first demonstrated by the plasminogen-specific EACA and AMCHA and extended here to MET antagonists.

The fragment-based approach adopted in this study appears to have considerable potential for targeting protein–protein interfaces where shallow groves or preformed small, but often deep, pockets are present and appear to have a role in the protein assembly. Protein–protein interfaces typically rely on shallow protein binding sites, but these often contain small well-defined pockets that recognise side chains or chain termini of proteins or peptides.^38^,^39^ This appears to have been true in work in which the Abbott Laboratories' targeted Bcl-XL, which inhibits apoptosis by binding the pro-apoptotic molecule BAK or another pro-apoptotic molecule BAD. Small molecules that mimic the alpha-helices involved in these interactions have been designed by growing from a concave hotspot to give molecules with affinities of up to 5 nM.^40^ In our own studies of the inhibition BRCA2 binding to Rad51, many fragments bind to the phenylalanine-binding pocket of Rad51 and these could be elaborated into molecules with nanomolar affinities.^41^

In the present study we have set the foundations for inhibiting MET signalling using fragments that bind into the *lysine-binding pocket* of the K1 domain of HGF/SF. We have identified a new chemical entity – MB605 – that binds NK1 with sub-millimolar affinity and displays favourable binding behaviour and activity in a cellular context. The next step is to grow MB605 into a high-affinity ligand and the structural and affinity data gained in this study can clearly guide the development of MB605 and its progress from hit to lead. MB605 is a very small molecule (140 Da) but displays about ten times better affinity for NK1 than HEPES and can be grown towards the glycine loop gaining the chemical space occupied by the ethylene glycol molecule in the current crystal structure (Fig. 4a) and enabling hydrogen bonding to the glycine loop with significant affinity gain.

## Materials and methods

### Protein production and purification

NK1 was expressed in methylotrophic *P. pastoris* and the purification was carried out in two steps as described previously by Chirgadze *et al.*,^19^ with one modification. MES buffer was removed from the purification process and 50 mM sodium phosphate, pH 7.4 was used instead. The SPH fragment (Val495-Ser728) was cloned from cDNA of full length human HGF/SF into the baculovirus secretion vector pAcGP67A (BD Biosciences) with the addition of a C-terminal hexahistidine tag and a C604S mutation. Viral stocks were amplified using Sf9 insect cells (BD Biosciences), and protein was produced using High Five™ (BTI-TN-5B1-4, Invitrogen) insect cells and ESF 921 (Expression Systems) media (+2.5% FCS). SPH domain was purified using a 5 mL HP HisTrap (GE Healthcare) column and a gradient of imidazole from 0–500 mM in 50 mM Tris, pH 7.7. Protein-containing fractions were pooled, concentrated and injected onto a 16/60 Superdex 200 prep grade column (Amersham Pharmacia Biotech) equilibrated with 10 mM HEPES, pH 7.2, 150 mM NaCl. Met567 was produced as previously described in Gherardi *et al.*^42^

### Piperazine-like compounds

1 M stock solutions of the piperazine-like compounds (Sigma) were prepared in water and their respective pHs were adjusted to a value within their buffering range by either HCl or NaOH. The seven piperazine-like compounds and their respective pHs are: HEPES (pH 7.6), (H)EPPS (pH 7.4), PIPES (pH 7.0), MES (pH 5.8), MOPS (pH 7.2), CHES (pH 8.6) and CAPS (pH 9.7). For the biological assay the pH of all the piperazine-like compounds was adjusted to 7.4, in order to match the pH of the media used to culture the cells in.

### Fragment library

The fragment library used in this study is an in-house library composed of 1338 compounds. Approximately 1200 of the compounds are from commercial libraries supplied by Maybridge (Thermo Fisher Scientific: http://www.maybridge.com/). In addition to the Maybridge set, the library includes approximately 100 compounds prepared in the Department of Chemistry, University of Cambridge in the laboratory of Prof Chris Abell, where it is maintained for screening a range of targets. All compounds are dissolved and stored in 100% DMSO. The library is not target-tailored. Details of library screening, results and statistics are given in the ESI† section of the manuscript.

### Kinetic measurements using SPR

Binding constants of piperazine-like compounds and fragments were determined using a Biacore T100 instrument. NK1 was immobilized on a CM5 chip by amine coupling to a density of 6000 RU. Compounds were stored as 100 mM stock solutions in water and serial dilutions were prepared in the running buffer (PBS pH 7.4, 0.05% Tween 20). The compound solutions were injected onto the surface of the chip at a flow rate of 30 μL min^–1^, contact time was 1 min and dissociation time 1 min. Sensorgrams were analysed using the Biacore T100 evaluation software and the binding constants determined.

### Kinetic measurements using NMR

Ligand screening was performed by monitoring the chemical shift variations in ^1^H-^15^N TROSY-HSQC NMR spectra of the uniformly [^15^N]-labelled NK1 protein (Fragai *et al.* 2015, *in prep.*) upon the addition of increasing concentrations of the investigated compounds.^43^–^45^ The experiments were performed using 0.18 mM protein samples in 25 mM Tris, 100 mM NaCl and 0.01% sodium azide, at pH 7.2 with 10% D_2_O. The spectra were recorded at 310 K on a *Bruker AVANCE* 900 MHz spectrometer equipped with a TXI cryo-probe. Sample solutions were prepared from 1 M or 100 mM DMSO stock solutions of the ligands. The dissociation constant values (*K*_D_) were calculated by plotting the weighted average ^1^H and ^15^N chemical shifts of the residues experiencing significant variations as a function of ligand concentration added during the titration and considering the one site binding model.^46^

### NK1 crystallization

NK1 buffered in 150 mM NaCl, 50 mM Tris, pH 7.5 (apo-NK1 and all soaking experiments) was concentrated to ∼20 mg mL^–1^ using a Vivaspin device with a 10 kDa cut-off membrane (Sartorius Biolab). Crystallisation was carried out in Linbro plates by the vapour diffusion hanging drop technique using a protein : precipitant ratio of 1 : 1 at 20 °C. Previously NK1 has been crystallised in 18% PEG 4000, 200 mM sodium acetate and 150 mM Tris pH 8.5 as precipitant.^19^ Here final crystallisation conditions (19% PEG 4000, 200 mM sodium acetate and 150 mM Tris pH 7.5) were optimised around those to achieve high-resolution apo structure. Details of compound soaking, X-ray data collection and structure determination are given in the ESI† section of the manuscript.

### Biological assays

The ability of MB605 and the piperazine-like compounds to inhibit MET, Erk and Akt phosphorylation in the presence of NK1 was tested using mammalian Vero cells as described by Ferraris *et al.*^47^ and further details are given in the ESI† section of the manuscript.

## Conclusions

Although targeting protein–protein interactions using a fragment-based approach unquestionably remains a major challenge, the data reported in the present study demonstrate that a major cancer target such as the HGF/SF-MET interface is amenable to this approach and provide both the building block for a small molecule MET antagonist and a roadmap for developing the MB605 compound into a full therapeutic.

## Accession codes

The atomic coordinates and structure factors for the reported crystal structures have been deposited with the Protein Data Bank (PDB) under accession code 5CS1 for the NK1 apo structure and for NK1 complexed with ligands under accession codes ; 5COE, ; 5CS3, ; 5CS5, ; 5CS9, ; 5CSQ, ; 5CT1, ; 5CT2, ; 5CT3 and ; 5CP9.

## Author contributions

A. G. S. designed the research, performed the experiments, analysed the data and wrote the paper. A. G. S., A. W. and T. L. B. developed methodology for the SPR fragment screening. A. W. carried out fragment screening on Met567 and wrote the paper. A. S. performed the phosphorylation assays. M. F. carried out the NMR measurements and analysed the data. D. C. provided support and guidance for crystallography. D. B. A. performed the GOLD docking and pharmacophore analysis. T. L. B. and E. G. conceived the project, guided the research, analysed data and wrote the paper.

## Supplementary Material

Supplementary information
