# Supplementary material for: Exploring the chemical space of the lysine-binding pocket of the first kringle domain of hepatocyte growth factor/scatter factor (HGF/SF) yields a new class of inhibitors of HGF/SF-MET binding
Source: Chem Sci. 2015 Jul 31;6(11):6147–57. doi: 10.1039/c5sc02155c (PMC6054100; doi:10.1039/c5sc02155c)
Supplement: Supplementary file 1 [file SC-006-C5SC02155C-s001.pdf]

Electronic Supplementary Material (ESI) for Chemical Science.
This journal is © The Royal Society of Chemistry 2015

# Exploring the chemical space of the lysine-binding pocket of the first kringle domain of hepatocyte growth factor/scatter factor (HGF/SF) yields a new class of inhibitors of HGF/SF-MET binding

## (Electronic Supplementary Information)

Sigurdardottir, A.G.[a,*,‡], Winter, A.[a,+], Sobkowicz, A.[b], Fragai, M.[c], Chirgadze, D.[a], Ascher D.B.[a], Blundell, T.L.[a,‡] and Gherardi, E.[b,d]

a Department of Biochemistry, University of Cambridge, 80 Tennis Court Road, Cambridge, CB2 1GA, UK

b Medical Research Council (MRC) Center, Hills Road, Cambridge CB2 0QH, UK.

c Magnetic Resonance Center (CERM) and Department of Chemistry, University of Florence, Via L. Sacconi 6, 50019 Sesto Fiorentino, Florence, Italy

d Unit of Immunology and General Pathology, Department of Molecular Medicine, University of Pavia, 9 via A Ferrata, 27100 Pavia, Italy

* Present address: MRC Laboratory of Molecular Biology, Francis Crick Avenue, Cambridge Biomedical Campus, Cambridge, CB2 0QH, UK

+ Present address: University of Leicester, Department of Biochemistry, Lancaster Road, LE2 9HN, UK

‡ Corresponding authors: Tom L. Blundell tlb20@cam.ac.uk

Anna G. Sigurdardottir sigurdar@mrc-lmb.cam.ac.uk

***Supplementary Materials and Methods***

**Fragment library screening and data analysis**

Met567 (soluble truncated fragment of the MET ectodomain, residues 25-567), NK1 and the SPH domain were immobilized on CM5 chips by amine coupling. NK1 was diluted into 10 mM phosphate pH 7.0 and the SPH domain into 10 mM acetate pH 5.5 to a final concentration of 2.5 μM and 1 μM, respectively, for coupling. The immobilization wizard within the Biacore T100 software was set to "aim for immobilized level" of 8000 RU to achieve complete saturation of the chip surface.

With minor deviations, the procedure and conditions outlined below were used for all screenings of an in-house fragment library of 1338 members against individual target proteins. The running buffer was PBS pH 7.4, 0.05% Tween 20, 0.25% DMSO (1% DMSO for Met567). Fragments were stored as 100 mM stock solutions in DMSO. Prior to screening, fragment samples were diluted to 500 μM (1 mM for Met567) in running buffer. Screening was performed in 96-well plates at a flow rate of 30 μl $min^{-1}$ with 1 min sample injection followed by dissociation for 1 min and surface regeneration using 1 M NaCl for 30 sec. After regeneration, SPR responses generally returned to base levels. However, if the post-regeneration baseline exceeded the pre-sample baseline by more than 10 RU, a second regeneration cycle with 50% ethylene glycol was used. In order to be able to correct for small variations in DMSO concentration between samples, eight solvent samples ranging from 0.012 to 1.5% DMSO were injected every 40th cycle. To monitor the binding capacity of the NK1, SPH and Met567 surfaces throughout the screening, injections of 500 μM HEPES (NK1), 0.25 μM Met567 (SPH) and 0.2 μM NK1 and 1% DMSO (Met567) were carried out every 40th cycle. The flow cell temperature was 25°C. NK1 and the SPH domain were immobilized on the same chip (channels 2 and 3 respectively) and were therefore screened in parallel. Met567 was screened separately and results are published in an accompanying paper (Winter *et al.* 2015, *in revision*).

For data analysis, first, all responses were reference and solvent corrected.[1] Next, equilibrium binding for every fragment was determined and hits established by applying several filters: sensorgram shape, upper and lower threshold. Typical sensorgrams from fragment screening show “square curves” in SPR due to the fast on and off rates and fragments not showing this response were discarded from further analysis. Binding levels are represented as response units (RU) but the SPR signal is a convolution of multiple parameters including target coupling density, target-binding activity, target molecular weight and molecular weight of each injected fragment. To be able to identify hits accurately and to be able to compare responses between different plates and targets, data were normalised against these factors. First the theoretical maximum response for each fragment was calculated using equation 1:

$$RU_{max} = \frac{Coupling\ density}{MW_{protein}} \times MW_{fragment} \qquad \text{(Equation 1)}$$

This $RU_{max}$ value differs for each protein target because it depends on the molecular weight of the protein and the amount of protein immobilized on the chip (coupling density). The $RU_{max}$ was then used to calculate the normalised relative SPR response:

$$Normalised\ relative\ SPR\ response = \frac{RU_{corrected}}{RU_{max}} \times 100\% \qquad \text{(Equation 2)}$$

which displays the binding observed compared to the theoretical maximum response a fragment should cause upon binding. 100% response would be 1:1 binding with levels above 100% implying over-stoichiometric binding.

**Compound soaking**

For this, 1 M stock solutions were prepared in DMSO (fragments) or water (piperazine-like compounds) and the pH adjusted to a value within their buffering range with either HCl or NaOH. The seven piperazine-like compounds and their respective pH are: HEPES (pH 7.6), (H)EPPS (pH 7.4), PIPES (pH 7.0), MES (pH 5.8), MOPS (pH 7.2), CHES (pH 8.6) and CAPS (pH 9.7). Secondary stocks of the compounds at 50 mM were prepared in the

respective mother liquor and soaked for up to 48 hours. In the case of CAPS, however, it was necessary to lower the pH of the stock solution to 7.4 and soak only for 8 hours to maintain crystal quality. MB605 was solubilised in 50% DMSO at 20 mM concentration (1% final DMSO concentration in the drop) in order to minimize adverse effects of DMSO on the protein crystal. 25% ethylene glycol was used as the cryoprotective agent.

### X-ray data collection and processing

X-ray datasets were collected either at the European Synchrotron Radiation Facility (ESRF, Grenoble, France), the Diamond Light Source (DLS, Oxford, UK) or in-house using the X8 PROTEUM (Bruker AXS) at the Department of Biochemistry, University of Cambridge. Datasets of at least 180° were collected. Raw data collected at synchrotrons were integrated, merged, scaled and reduced using DENZO and SCALEPACK.[2] Raw data collected in-house were integrated, merged, scaled and reduced using the PROTEUM 2 software package.[3] The CCP4 suite of crystallographic programs (Collaborative Computational Project 1994) was used for all subsequent steps of the structure determination.[4,5] The structure of NK1 was originally solved by combination of molecular replacement and non-crystallographic symmetry averaging to a resolution of 2.5 Å (PDB: 1NK1).[6] Because the crystals were in the same monoclinic space group $P2_1$ as the 1NK1 structure and the unit cell parameters were comparable, the previous structure could be used as a starting point for the refinement of the crystal structure. The sigmaA weighted 2Fo-Fc and Fo-Fc electron density maps were calculated to allow rebuilding and refitting of the model using Coot.[7] Figures were prepared using PyMOL.[8]

## Molecular docking

All small molecules were constructed using standard bond lengths and bond angles and then geometrically optimized within SYBYL-X 2.0 (Certara, L. P., http://www.tripos.com) using the MMFF94s forcefield and partial atomic charges, conjugate gradient convergence method. Termination of the optimization was achieved when the gradient difference of successive steps was < 0.05 kcals $mol^{-1}$ Å. The side chains of NK1 (PDB: 1NK1) after removing all ligands were geometry optimized for 1000 iterations (or until the gradient of successive iterations was < 0.05 kcal $mol^{-1}$ Å) using the same protocol as above. The compounds were then docked into the NK1 structure using the docking algorithm Surflex (within SYBYL-X 2.0, Certara L. P., http://tripos.com). The protocol was generated by manually selecting the residues lining the pocket, a threshold of 0.50 and a bloat value of 2. The docking mode used was GeomX and protein flexibility was allowed, all other parameters were at default values. The scoring function used was C-Score and the top 100 ranked poses of each inhibitor were retained for examination. The top five conformations showed minimal deviation from the conformations of the compounds observed crystallography in complex with the NK1 dimer. The top conformations were then used to explore the pharmacophore properties of the NK1 pocket based upon their potential interactions within the pocket.

## Biological Assays

For MET, Erk1/2 and Akt phosphorylation assays, Vero cells were seeded at $2.1 \times 10^5$ cells $well^{-1}$ (2 ml $well^{-1}$) in 6-well plates and grown to 90% confluence in 10% FCS/DMEM media and then switched to serum-free medium (to avoid any growth factors present in the serum). 24 hours post starvation the cells were stimulated for 5 min at 37°C with each individual piperazine-like compounds as well as with NK1 (1 nM) pre-incubated with different concentrations of the piperazine-like compounds (100 mM, 10 mM, 1 mM and 100 µM) or MB605 (1 mM, 100 µM and 10 µM). Stimulations with either 0.1% DMSO or 1 nM of NK1

solution were used as a negative and a positive control, respectively. The cells were then washed in PBS buffer, lysed on ice for 30 min and frozen at -80ºC until further analysis. For Western blotting experiments, equal amounts of lysates were loaded onto 10% SDS-PAGE gels. Proteins were then transferred onto a nitrocellulose membrane and analysed by using antibodies from New England Biolabs (UK) Ltd, Hitchin, (Cell Signaling) unless otherwise stated: rabbit antiphospho-Met (Tyr1234/1235) (D26) XPTM antibody (c/n3077); mouse anti-Met (L41G3) antibody (c/n3148); rabbit anti-phospho-Akt (Ser473) antibody (c/n9271); rabbit anti-Akt (c/n9272); mouse anti-phospho-Erk antibody (Sigma M8159); rabbit anti-Erk1/2 (Promega, V114). HRP-conjugated secondary antibodies, goat anti-rabbit IgG (Dako, Ely, UK) and rabbit anti-mouse IgG (Dako) were used for 1 hour incubation of the membrane at RT, and blots were developed with SuperSignal® West Pico Chemiluminescent Substrate (Thermo Scientific, UK).

***Supplementary Results***

**Fragment library screening to identify small molecule binders**

SPR was used to screen a fragment library of 1338 compounds against NK1, SPH and MET immobilized on CM5 chips. Almost all protein surfaces showed a loss of the active fraction with time, most likely due to unfolding hence this process and the chip response was monitored and controlled throughout the screenings, as outlined in Supplementary Materials and Methods. NK1 and the SPH domain were screened in parallel, i.e. on the same chip at a fragment concentration of 500 μM, Met567 was screened at 1 mM (Winter *et al.* 2015, *in revision*). These concentrations are within the range (0.1 to 1.0 mM) typically used in primary screens.[9] (Higher screening concentrations typically result in a larger signal, making weakly interacting compounds easier to detect but also increase the probability of non-specific binding and aggregation).

Response levels from SPR were normalised to the molecular weights of the fragments and calculated as percent response of maximum expected response. Over-stoichiometric binders or aggregating fragments were easily identified by their extremely high responses. Sensorgram shape was inspected and two thresholds were employed to exclude undesired binding behaviour (sensorgram shape), non-binders (low threshold of 25%) and potentially aggregating fragments (upper threshold of 300%). At this stage 828 fragments remained in the hit-pool for NK1. We defined promiscuous binders as those molecules that show binding to all three proteins: NK1, Met567 and the SPH domain. A hit was defined as a fragment that showed clear selectivity for NK1 over the other two proteins and, after employing the selectivity filter, 71 hits were confirmed for NK1 (Fig. S4a), which gives a hit rate of 5% for NK1. Fragment hits were mostly small heterocyclic compounds that could be further classified into five main scaffolds: 1) 23 fragments with a common benzene ring, 2) 12 fragments were 6-membered heterocyclic derivatives, 3) 22 fragments were 5-membered heterocyclic derivatives, 4) seven fragments were bicyclic derivatives, and 5) seven compounds had two connected rings (ESI Tables S3-7). These data are encouraging and

indicate that SPR could find wide applicability for screening protein-protein interfaces. An overview of the complete screening and data analysis strategy can be seen in Fig. S4a.

Steady-state binding constants, $K_D$, were determined using SPR, and were mostly in the millimolar range which is not unusual for small molecules. 11 fragments (15%) did not show any binding in this secondary screen despite being identified as positive hits in the first screening; these were assumed to be false positives and excluded from further analysis (see Fig. S4b). All 11 fragments ranked last in the fragment screening, suggesting that the selection criteria for a hit (the lower threshold) had been too low. 28 fragments or nearly 40% of the initial fragment hits did not show the expected binding curves but instead a linear concentration dependency (resulting in $K_D$ > 1000 mM); they were therefore rejected from further analysis. This concentration-independent behaviour might be due to low solubility, aggregation or unwanted interaction with the chip surface. However, 24 fragments (34%) showed good binding behaviour and affinity better than 3 mM. These fragments and can be considered as validated hits for NK1.

***Supplementary Figures***

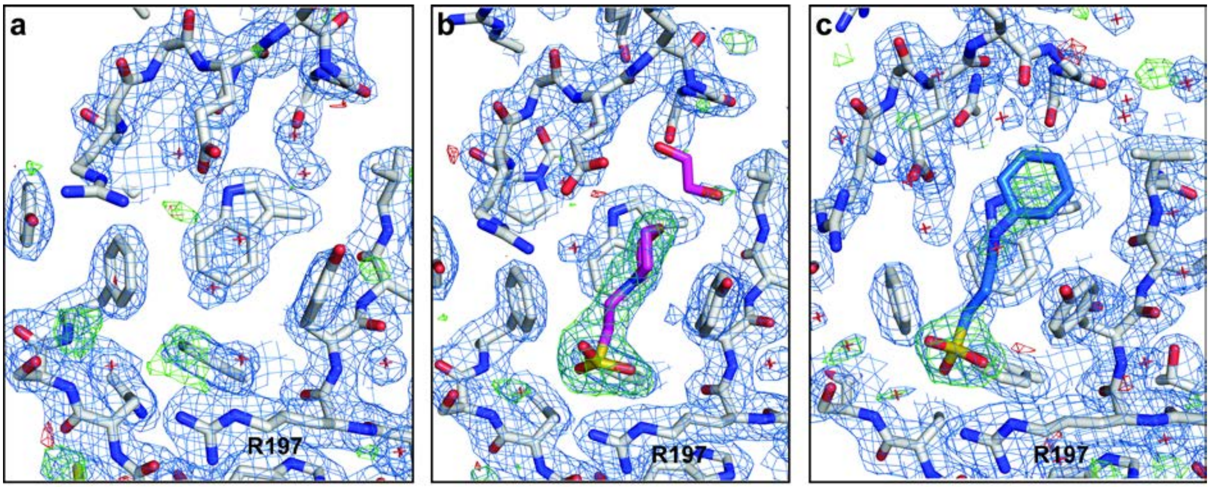


**Fig. S1** Representative examples of the electron density of the *lysine-binding pocket* for NK1 structures in complex with piperazine-like compounds. (a) NK1 apo structure (PDB ID: 5CS1). NK1 in complex with (b) MES (magenta, PDB ID: 5CS9) and (c) CAPS (light blue, PDB ID: 5CT2). The maps shown are calculated without the ligand present (i.e. final round of refinement before inserting the ligand) and the model displayed is the final refined model. The 2Fo-Fc map (blue) is contoured at 1.0 σ and the Fo-Fc map at 3.0 σ. Protein carbon atoms are gray, oxygen atoms are in red and nitrogen atoms are in blue. Water molecules are red crosses.

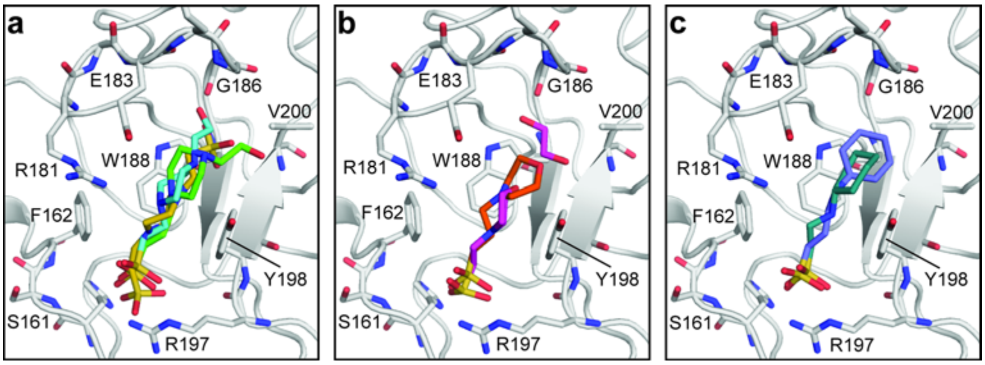


**Fig. S2** Overlay of the piperazine-like compounds binding in the *lysine-binding pocket* of NK1. (a) HEPES (cyan), (H)EPPS (green) and PIPES (yellow). (b) MES (magenta) and MOPS (orange). (c) CHES (blue green) and CAPS (light blue). Side chain atoms of residues involved in binding the compounds and the conserved water molecule (dark red) are shown. Protein carbon atoms are gray, oxygen atoms are in red and nitrogen atoms are in blue.

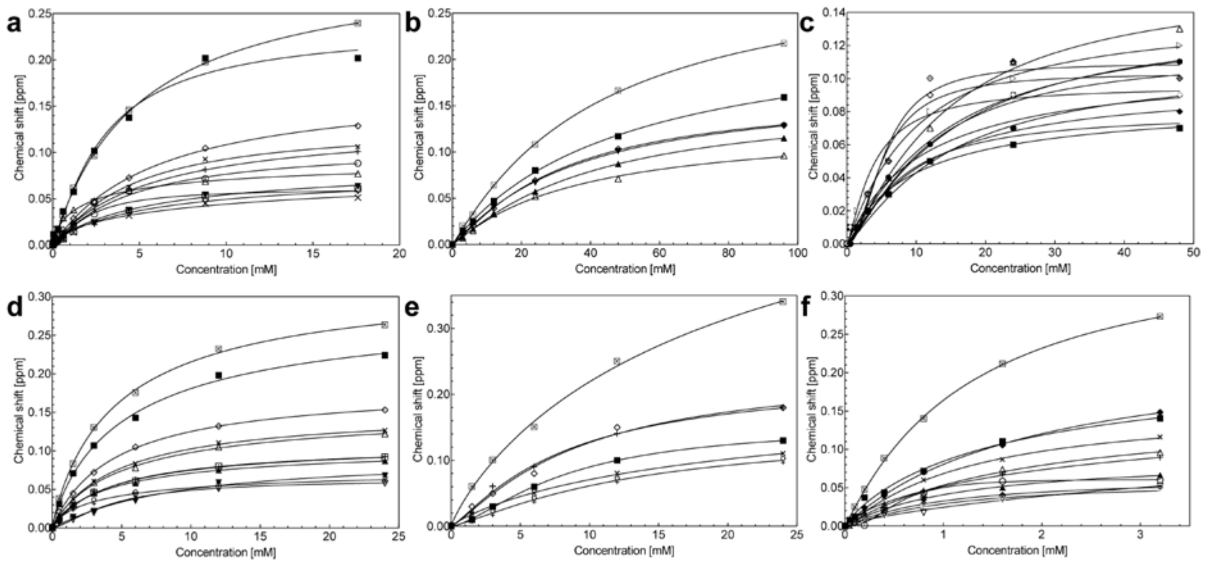


**Fig. S3** Binding curves of the residues that experience the largest chemical shift in 2D $^{1}$H-$^{15}$N HSQC NMR spectroscopy upon binding of the piperazine-like compounds to NK1. (a) **(H)EPPS** – R178 (×), R181 (○), G182 (**+**), E183 (▼), E184 (△), G185 (■), G186 (✖), W188 (□), Y198 (☒), E199 (◇) and C201 (▽). (b) **PIPES** – E183 (▼), E184 (△), G185 (■), Y198 (☒), E199 (◇) and V200 (▲). (c) **MES** – H40 (◈), H158 (✦), E159 (✧), S161 (●), G182 (**+**), E184 (△), G185 (■), G186 (✖), S192 (▷), V196 (⬢), R197 (◆), Y198 (☒) and E199 (◇). (d) **MOPS** – N179 (♀), G182 (**+**), E183 (▼), E184 (△), G185 (■), G186 (✖), W188 (□), Y198 (☒), E199 (◇), V200 (▲) and C201 (▽). (e) **CHES** – N179 (♀), G182 (**+**), G185 (■), G186 (✖), Y198 (☒) and E199 (◇). (f) **CAPS** – R181 (○), G182 (**+**), E184 (△), G185 (■), G186 (✖), C189 (⊗), R197 (◆), Y198 (☒), E199 (◇), V200 (▲) and C201 (▽).

**a**

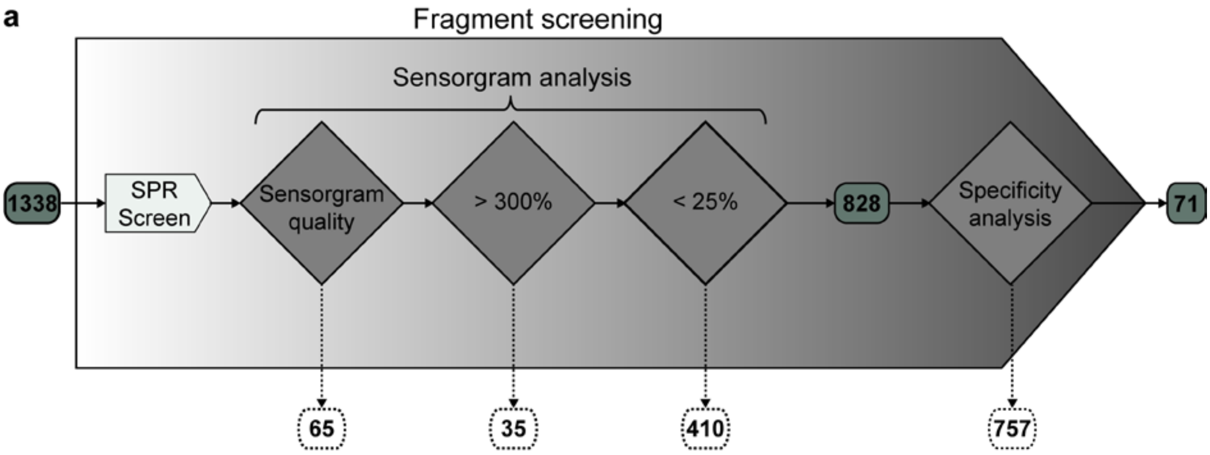


**b**

| Reason for elimination from initial hit list | Number of fragments | Portion of initial hit list | Portion of initial fragment library |
|---|---|---|---|
| No binding | 11 | 15% | 0.8% |
| Linear concentration dependency | 28 | 40% | 2.1% |
| Affinity lower than 3 mM | 8 | 11% | 0.6% |
| Final hit list | 24 | 34% | 1.8% |

**Fig. S4** Overview of the strategy used for fragment screening and hit validation. (a) During the screening campaign fragments were rejected on the basis of sensorgram quality, lower and upper binding thresholds and specificity to NK1 versus Met567 and SPH. Numbers in dark gray squares refer to the number of fragments selected for the following step and those in dashed squares show the number of excluded fragments. Arrows represent experimental assay, while diamonds represent analysis steps. (b) The validation process eliminated poor binders and unsuitable fragments to give a final hit list of 24 fragments.

# Supplementary Tables

**Table S1** Crystallographic dataset statistics for NK1 in complex with piperazine-like compounds and MB605.

| | **NK1_apo** | **HEPES** | **(H)EPPS** | **PIPES** | **MES** | **MOPS** | **CHES** | **CAPS** | **2FA** | **MB605** |
|---|---|---|---|---|---|---|---|---|---|---|
| **PDB accession codes** | **5CS1** | **5COE** | **5CS3** | **5CS5** | **5CS9** | **5CSQ** | **5CT1** | **5CT2** | **5CT3** | **5CP9** |
| **Resolution limits (Å) (last shell)** | 50.0 – 2.0 (2.05 – 2.00) | 50.0 – 2.2 (2.25 – 2.20) | 34.3 – 2.5 (2.59 – 2.50) | 50.0 – 1.9 (1.94 – 1.90) | 50.0 – 2.0 (2.05 – 2.00) | 50.0 –1.95 (2.00 – 1.95) | 50.0 – 2.0 (2.05 – 2.00) | 32.2 – 2.0 (2.10 – 2.00) | 30.0 – 2.0 (2.05-2.00) | 50.0 – 1.9 (1.94 – 1.90) |
| **Space group** | $P2_1$ | $P2_1$ | $P2_1$ | $P2_1$ | $P2_1$ | $P2_1$ | $P2_1$ | $P2_1$ | $P2_1$ | $P2_1$ |
| **Unit cell parameters** | a = 53.39 Å | a = 53.28 Å, | a = 53.34 Å | a = 53.78 Å | a = 53.29 Å | a = 53.68 Å | a = 53.83 Å | a = 54.10 Å | a = 53.23 Å, | a = 53.39 Å, |
| | b = 63.40 Å | b = 63.08 Å, | b = 63.38 Å | b = 63.63 Å | b = 64.15 Å | b = 63.53 Å | b = 63.58 Å | b = 63.44 Å | b = 63.08 Å, | b = 63.65 Å, |
| | c = 57.62 Å | c = 57.38 Å, | c = 57.48 Å | c = 57.40 Å | c = 57.20 Å | c = 57.53 Å | c = 57.33 Å | c = 57.36 Å | c = 57.28 Å, | c = 57.20 Å, |
| | β = 94.84° | β = 95.04° | β = 95.17° | β = 95.36° | β = 94.96° | β = 95.39° | β = 95.62° | β = 95.87° | β = 95.06° | β = 95.25° |
| **Unique reflections** | 26,001 | 19,668 | 13,345 | 30,379 | 26,007 | 27,756 | 25,839 | 26,231 | 25,692 | 30,034 |
| **Redundancy (last shell)** | 3.6 (3.2) | 3.4 (2.8) | 7.2 (5.1) | 3.7 (3.7) | 4.7 (4.6) | 3.7 (3.7) | 3.7 (3.7) | 26.3 (12.4) | 3.5 (3.3) | 3.7 (3.7) |
| **$R_{sym}$[1)] (%) (last shell)** | 5.2 (30.3) | 5.4 (37.4) | 12.7 (52.8) | 4.4 (25.9) | 7.0 (45.0) | 4.0 (31.1) | 4.7 (38.1) | 11.0 (78.0) | 5.5 (39.8) | 4.0 (25.9) |
| **Completeness (%) (last shell)** | 99.5 (96.4) | 99.6 (97.7) | 99.9 (99.9) | 99.3 (99.9) | 99.4 (99.8) | 98.7 (99.8) | 99.6 (99.9) | 99.9 (99.9) | 99.8 (99.8) | 99.4 (99.8) |
| **$\langle I/\sigma(I) \rangle$** | 12.5 | 14.6 | 9.7 (1.8) | 18.6 | 12.8 | 20.5 | 14.9 | 21.4 (1.6) | 16.1 | 20.1 |
| **Reflections with $\langle I/\sigma(I) \rangle$ >3 (%) (last shell)** | 81.4 (47.0) | 77.6 (36.3) | N/A[*] | 85.7 (54.6) | 81.7 (42.9) | 85.0 (48.2) | 80.2 (43.2) | N/A[*] | 81.8 (43.3) | 86.5 (56.6) |

[1)] $R_{sym} = \sum_h | I_h - \langle I \rangle | / \sum_h I_h$ , where $I_h$ is the intensity of reflection h and $\langle I \rangle$ is the mean intensity of all symmetry-related reflections.

[*)] Not included in statistical analysis in PROTEUM 2 software package.[3]

**Table S2** Refinement statistics for NK1 in complex with piperazine-like compounds and MB605.

| PDB accession codes | NK1_apo<br>5CS1 | HEPES<br>5COE | (H)EPPS<br>5CS3 | PIPES<br>5CS5 | MES<br>5CS9 | MOPS<br>5CSQ | CHES<br>5CT1 | CAPS<br>5CT2 | 2FA<br>5CT3 | MB605<br>5CP9 |
|---|---|---|---|---|---|---|---|---|---|---|
| **Resolution limits (Å)** | 42.6 – 2.0 | 40.6 – 2.2 | 34.3 – 2.5 | 26.1 – 1.9 | 26.1 – 2.0 | 26.3 – 1.95 | 26.0 – 2.0 | 32.2 – 2.0 | 26.5 – 2.0 | 26.6 – 1.9 |
| **Total reflections (test for $R_{free}$)** | 24,664 (1,323) | 18,649 (1,007) | 12,667 (657) | 28,834 (1,528) | 24,666 (1,323) | 26,352 (1,389) | 24,511 (1,308) | 24,333 (1,299) | 24,347 (1,303) | 28,491 (1,524) |
| **Total protein atoms (residues)** | 2,759 (346) | 2,812 (346) | 2,782 (346) | 2,783 (346) | 2,768 (346) | 2,774 (346) | 2,779 (346) | 2,774 (346) | 2,774 (346) | 2,780 (346) |
| **Total ligand atoms (molecules)** | - | 15 (1) | 16 (1) | 18 (1) | 16 (2) | 26 (2) | 13 (1) | 28 (2) | 8 (1) | 14 (2) |
| **Water molecules** | 168 | 137 | 91 | 282 | 155 | 153 | 150 | 194 | 191 | 234 |
| **$R_{cryst}$ [1] (%)** | 21.1 | 20.7 | 21.3 | 19.6 | 21.3 | 22.3 | 21.7 | 22.7 | 20.5 | 19.5 |
| **$R_{free}$ [2] (%)** | 25.5 | 26.4 | 31.8 | 24 | 25.1 | 27.4 | 26.9 | 29.5 | 25.1 | 22.7 |
| **Average B-factor ($Å^2$)** | 36.3 | 44.3 | 39.2 | 35.1 | 42.8 | 43.8 | 40.1 | 34.2 | 37.0 | 36.1 |
| **Ramachandran plot analysis, number of residues in:** | | | | | | | | | | |
| **Favoured regions (%)** | 93.8 | 93.7 | 89.4 | 94.1 | 93.5 | 93.6 | 95.0 | 95.3 | 95.0 | 94.7 |
| **Allowed regions (%)** | 5.3 | 5.4 | 8.5 | 5.3 | 6.2 | 5.9 | 5.0 | 4.1 | 4.4 | 4.4 |
| **Disallowed regions (%)** | 0.9 | 0.9 | 2.1 | 0.6 | 0.3 | 0.6 | 0.0 | 0.6 | 0.6 | 0.9 |
| **R.m.s. deviations:** | | | | | | | | | | |
| **Bond lengths (Å)** | 0.010 | 0.013 | 0.017 | 0.009 | 0.011 | 0.011 | 0.012 | 0.011 | 0.010 | 0.009 |
| **Bond angles (°)** | 1.23 | 1.38 | 1.83 | 1.15 | 1.47 | 1.23 | 1.40 | 1.32 | 1.24 | 1.15 |

[1] $R_{cryst} = (\sum |F_o - F_c|) / \sum F_o$ , where $F_o$ and $F_c$ are observed and calculated structure factor amplitudes.

[2] $R_{free}$ as for $R_{cryst}$ using a random subset of the data (5%) not included in the refinement.

**Table S3** NK1 fragment hits – Benzene derivatives.

**a) Carbonyl derivatives**

| Compound name | Structure | SPR response [%][1)] | Ranking order[2)] | $K_D$ [mM][3)] |
|---|---|---|---|---|
| **MB1306** | 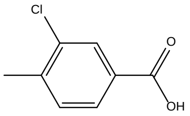 | 77 | **21** | 2.5 |
| **MB1318** | 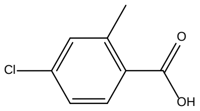 | 61 | **33** | 0.36 |
| **MB1300** | 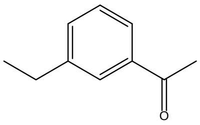 | 93 | **14** | >1000 |
| **MB1315** | 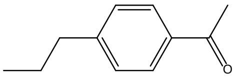 | 52 | **47** | 0.94 |
| **MB1174** | 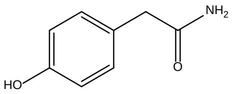 | 43 | **53** | >1000 |
| **MB1284** | 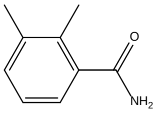 | 90 | **15** | 0.86 |
| **MB1321** | 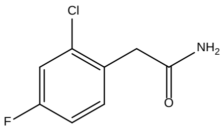 | 65 | **28** | >1000 |
| **MB1267** | 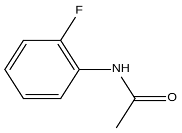 | 90 | **16** | 1.6 |

[1)] Normalised relative value against NK1.
[2)] Based on fragment screening.
[3)] Steady-state binding constant from SPR.

**Table S3 continued** NK1 fragment hits – Benzene derivatives.

**b) Amino derivatives**

| Compound name | Structure | SPR response [%][1] | Ranking order[2] | $K_D$ [mM][3] |
|---|---|---|---|---|
| **MB1274** | 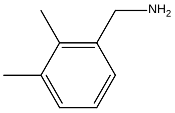 | 105 | **7** | 1.6 |
| **MB396** | 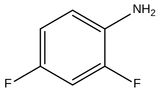 | 83 | **18** | 1.1 |
| **MB409** | 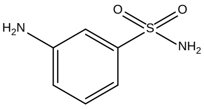 | 36 | **59** | >1000 |

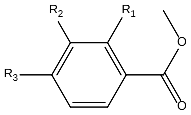


**c) Methyl benzoates**

| Compound name | $R_1$ | $R_2$ | $R_3$ | SPR response [%][1] | Ranking order[2] | $K_D$ [mM][3] |
|---|---|---|---|---|---|---|
| **MB1253** | –F | –H | –H | 98 | **11** | 2.9 |
| **MB1301** | –Cl | –H | –F | 70 | **25** | >1000 |
| **MB1307** | –H | –Cl | –$CH_3$ | 62 | **31** | >1000 |
| **MB1292** | –$OCF_3$ | –H | –H | 63 | **30** | >1000 |

1) Normalised relative value against NK1.
2) Based on fragment screening.
3) Steady-state binding constant from SPR.

**Table S3 continued** NK1 fragment hits – Benzene derivatives.

**d) Tri-/Difluoro derivatives**

| Compound name | Structure | SPR response [%][1)] | Ranking order[2)] | $K_D$ [mM][3)] |
|---|---|---|---|---|
| **MB1251** | 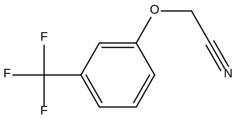 | 66 | **27** | >1000 |
| **MB406** | 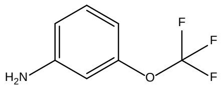 | 42 | **54** | >1000 |
| **MB1100** | 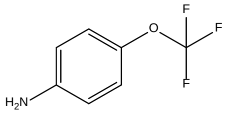 | 27 | **70** | #[4)] |
| **MB1291** | 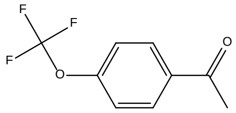 | 68 | **26** | >1000 |
| **MB1293** | 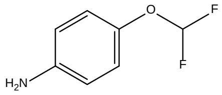 | 72 | **22** | 5.0 |

**e) Others**

| Compound name | Structure | SPR response [%][1)] | Ranking order[2)] | $K_D$ [mM][3)] |
|---|---|---|---|---|
| **MB894** | 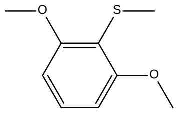 | 49 | **51** | >1000 |
| **MB1283** | 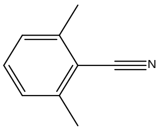 | 94 | **13** | >1000 |
| **MB1308** | 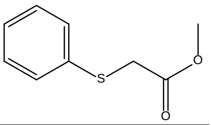 | 60 | **34** | >1000 |

[1)] Normalised relative value against NK1.
[2)] Based on fragment screening.
[3)] Steady-state binding constant from SPR.
[4)] Unable to measure the binding constant.

**Table S4** NK1 fragment hits – 6-membered heterocyclic derivatives

**a) 6-membered saturated rings**

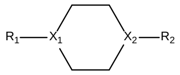


| Compound name | $X_1$ | $X_2$ | $R_1$ | $R_2$ | SPR response [%][1)] | Ranking order[2)] | $K_D$ [mM][3)] |
|---|---|---|---|---|---|---|---|
| **MB1129** | N | ‡[4)] | –H | –COOH | 30 | **67** | #[5)] |
| **MB1065** | O | ‡ | ‡ | $–CON(CH_3)_2$ | 38 | **57** | >1000 |
| **MB1082** | O | N | ‡ | $–C(S)NH_2$ | 53 | **43** | 0.37 |
| **MB1016** | ‡ | ‡ | $–NH_2$ | $–CH_2CH_3$ | 35 | **61** | # |

**b) Pyridines and pyrimidines**

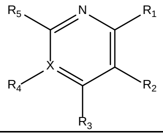


| Compound name | X | $R_1$ | $R_2$ | $R_3$ | $R_4$ | $R_5$ | SPR response [%][1)] | Ranking order[2)] | $K_D$ [mM][3)] |
|---|---|---|---|---|---|---|---|---|---|
| **MB404** | ‡[4)] | –H | –H | $–NH_2$ | –H | –H | 118 | **5** | 2.8 |
| **MB1003** | ‡ | –H | –H | $–CH_2NHCH_3$ | –H | –H | 107 | **6** | 6.4 |
| **MB1290** | ‡ | –H | $–CH_2NHCH_3$ | –H | –H | –H | 102 | **9** | 1.64 |
| **MB1317** | ‡ | –H | $–CH_2NHCOCH_3$ | –H | –H | –H | 60 | **35** | >1000 |
| **MB389** | ‡ | $–CH_2OH$ | –H | –H | –H | $–CH_3$ | 72 | **23** | 4.32 |
| **MB1261** | ‡ | –H | –H | –H | –H | $–OCH_3$ | 95 | **12** | 0.35 |
| **MB1260** | ‡ | $–CH_3$ | –H | –H | –CN | –O | 105 | **8** | 5.6 |
| **CA023** | N | –H | –H | –H | ‡ | $–NH_2$ | 52 | **46** | 0.77 |

1) Normalised relative value against NK1.
2) Based on fragment screening.
3) Steady-state binding constant from SPR.
4) Does not apply for this compound.
5) Unable to measure the binding constant

**Table S5** NK1 fragment hits – 5-membered heterocyclic derivatives

**a) 5-membered saturated rings**

| Compound name | Structure | SPR response [%][1)] | Ranking order[2)] | $K_D$ [mM][3)] |
|---|---|---|---|---|
| **MB975** | 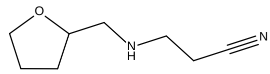 | 33 | **65** | #[4)] |
| **MB1017** | 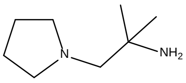 | 26 | **71** | # |

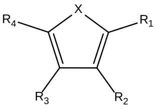


**b) 5-membered unsaturated rings**

| Compound name | X | $R_1$ | $R_2$ | $R_3$ | $R_4$ | SPR response [%][1)] | Ranking order[2)] | $K_D$ [mM][3)] |
|---|---|---|---|---|---|---|---|---|
| **MB915** | S | $–NH_2$ | –CN | –H | –H | 71 | **24** | 1.6 |
| **MB1237** | S | $–CH_2NH_2$ | $–CH_3$ | –H | –H | 118 | **4** | 4.2 |
| **MB914** | S | $–C(CH_3)_3$ | –H | $–NH_2$ | –C(O)OCH3 | 34 | **63** | #[4)] |
| **MB916** | S | $–C(O)OCH_3$ | –Cl | $–CH_3$ | –H | 34 | **64** | # |
| **MB921** | S | –H | $–CH_3$ | –H | –COOH | 49 | **52** | >1000 |
| **MB1217** | S | $–SCH_3$ | –H | –H | –COOH | 50 | **49** | >1000 |
| **MB605** | O | –H | –H | –H | $–CH_2CH_2COOH$ | 215 | **1** | 0.31 |

1) Normalised relative value against NK1.

2) Based on fragment screening.

3) Steady-state binding constant from SPR.

4) Unable to measure the binding constant

**Table S5 continued** NK1 fragment hits – 5-membered heterocyclic derivatives

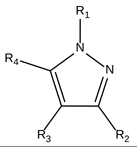


**c) Pyrazoles**

| Compound name | $R_1$ | $R_2$ | $R_3$ | $R_4$ | SPR response [%][1)] | Ranking order[2)] | $K_D$ [mM][3)] |
|---|---|---|---|---|---|---|---|
| **MB936** | –H | $–CH_3$ | –H | $–NH_2$ | 56 | **39** | 30 |
| **MB935** | $–CH_3$ | $–CH_3$ | –H | $–NH_2$ | 54 | **41** | 1.0 |
| **MB893** | $–CH_3$ | $–CH_3$ | $–NH_2$ | $–CH_3$ | 59 | **36** | 1.5 |
| **MB925** | –H | $–CF_3$ | –H | –H | 52 | **48** | 1.3 |
| **MB926** | $–CH_3$ | $–CF_3$ | –H | –H | 30 | **66** | #[4)] |

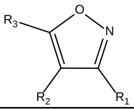


**d) Isoxazoles**

| Compound name | $R_1$ | $R_2$ | $R_3$ | SPR response [%][1)] | Ranking order[2)] | $K_D$ [mM][3)] |
|---|---|---|---|---|---|---|
| **MB1299** | $–CH_3$ | –H | $–CH_3$ | 131 | **2** | 2.2 |
| **MB1136** | $–CH_3$ | $–CH_3$ | $–NH_2$ | 39 | **55** | >1000 |
| **MB397** | $–NH_2$ | –H | $–CH_3$ | 81 | **20** | >1000 |

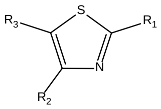


**e) Thiazoles**

| Compound name | $R_1$ | $R_2$ | $R_3$ | SPR response [%][1)] | Ranking order[2)] | $K_D$ [mM][3)] |
|---|---|---|---|---|---|---|
| **MB996** | –H | $–CH_3$ | –COOH | 38 | **56** | >1000 |
| **MB887** | $–CH_3$ | $–C(CH_3)_3$ | –H | 62 | **32** | >1000 |
| **MB895** | $–CH_3$ | $–CH_2COOH$ | –H | 53 | **45** | 0.43 |
| **MB1250** | $–CH_3$ | –CN | –H | 126 | **3** | 1.8 |
| **MB1252** | $–CH_3$ | $–C(S)NH_2$ | –H | 101 | **10** | 5.2 |

1) Normalised relative value against NK1.

2) Based on fragment screening.

3) Steady-state binding constant from SPR.

4) Unable to measure the binding constant

**Table S6** NK1 fragment hits – Bicyclic derivatives

**a) Fusion across a bond between two atoms**

| **Compound name** | **Structure** | **SPR response [%][1]** | **Ranking order[2]** | **$K_D$ [mM][3]** |
|---|---|---|---|---|
| **AT0381** | 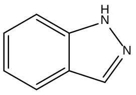 | 53 | **44** | 0.74 |
| **AT0541** | 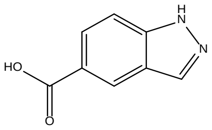 | 56 | **38** | >1000 |
| **MB415** | 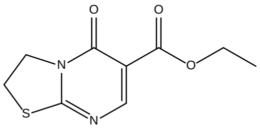 | 37 | **58** | >1000 |
| **MB417** | 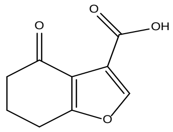 | 87 | **17** | 0.42 |
| **MB1058** | 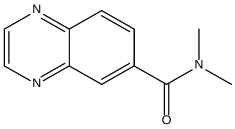 | 63 | **29** | >1000 |

**b) Fusion across a sequence of atoms (bridgehead)**

| **Compound name** | **Structure** | **SPR response [%][1]** | **Ranking order[2]** | **$K_D$ [mM][3]** |
|---|---|---|---|---|
| **MB385** | 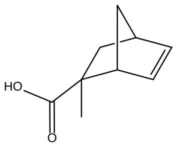 | 54 | **42** | >1000 |
| **MB405** | 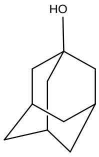 | 56 | **40** | >1000 |

[1] Normalised relative value against NK1.

[2] Based on fragment screening.

[3] Steady-state binding constant from SPR.

**Table S7** NK1 fragment hits – Two connected rings

**a) Biaryl compounds**

| Compound name | Structure | SPR response [%][1)] | Ranking order[2)] | $K_D$ [mM][3)] |
|---|---|---|---|---|
| **MB414** | 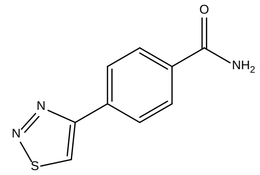 | 50 | **50** | >1000 |
| **MB900** | 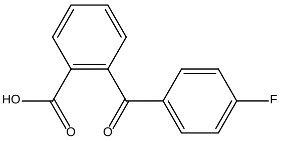 | 28 | **69** | #[4)] |
| **MB1236** | 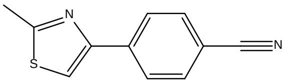 | 83 | **19** | 1.2 |

**b) Two connected rings**

| Compound name | Structure | SPR response [%][1)] | Ranking order[2)] | $K_D$ [mM][3)] |
|---|---|---|---|---|
| **MB1015** | 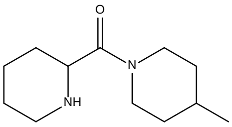 | 35 | **60** | >1000 |
| **MB1066** | 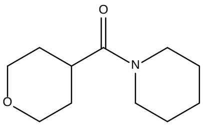 | 34 | **62** | #[4)] |
| **MB1067** | 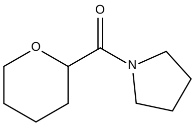 | 28 | **68** | # |
| **MB1218** | 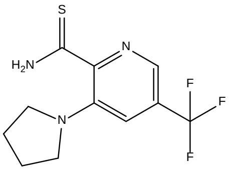 | 57 | **37** | 17.3 |

[1)] Normalised relative value against NK1.

[2)] Based on fragment screening.

[3)] Steady-state binding constant from SPR.

[4)] Unable to measure the binding constant

***REFERENCES***


1. Å. Frostell-Karlsson, A. Remaeus, H. Roos, K. Andersson, P. Borg, M. Hämäläinen and R. Karlsson, *J. Med. Chem.*, 2000, **43**, 1986-1992.
2. Z. Otwinowski and W. Minor, *Methods Enzymol.*, 1996, **276**, 301-315.
3. PROTEUM2, Version 2010, Bruker AXC Inc., Madison, Wisconsin, USA
4. G. N. Murshudov, A.A.Vagin and E. J. Dodson, *Acta Crystallogr D*, 1997, **53**, 240-255.
5. M. Winn, C. Ballard, K. Cowtan, E. Dodson, P. Emsley, P. Evans, R. Keegan, E. Krissinel, A. Leslie, A. McCoy, S. McNicholas, G. Murshudov, N. Pannu, E. Potterton, H. Powell, R. Read, A. Vagin and K. Wilson, *Acta Crystallogr D*, 2011, **67**, 235-242.
6. D. Y. Chirgadze, J. P. Hepple, H. Zhou, R. A. Byrd, T. L. Blundell and E. Gherardi, *Nat. Struct. Biol.*, 1999, **6**, 72-79.
7. P. Emsley, B. Lohkamp, W. G. Scott and K. Cowtan, *Acta Crystallogr D*, 2010, **66**, 486-501.
8. The PyMOL Molecular Graphics System, Version 1.7.4 Schrödinger, LLC.
9. A. M. Giannetti, *Methods Enzymol.*, 2011, **493**, 169-218.
